# Comparative population genomics identified genomic regions and candidate genes associated with fruit domestication traits in peach

**DOI:** 10.1111/pbi.13112

**Published:** 2019-04-16

**Authors:** Ke Cao, Yong Li, Cecilia H. Deng, Susan E. Gardiner, Gengrui Zhu, Weichao Fang, Changwen Chen, Xinwei Wang, Lirong Wang

**Affiliations:** ^1^ The Key Laboratory of Biology and Genetic Improvement of Horticultural Crops (Fruit Tree Breeding Technology) Ministry of Agriculture Zhengzhou Fruit Research Institute Chinese Academy of Agricultural Sciences Zhengzhou China; ^2^ The New Zealand Institute for Plant & Food Research Limited (PFR) Mount Albert Research Centre Auckland New Zealand; ^3^ The New Zealand Institute for Plant & Food Research Limited (PFR) Palmerston North Research Centre Palmerston North New Zealand

**Keywords:** population genomics, evolution, genome‐wide association studies, fruit weight, peach

## Abstract

Crop evolution is a long‐term process involving selection by natural evolutionary forces and anthropogenic influences; however, the genetic mechanisms underlying the domestication and improvement of fruit crops have not been well studied to date. Here, we performed a population structure analysis in peach (*Prunus persica*) based on the genome‐wide resequencing of 418 accessions and confirmed the presence of an obvious domestication event during evolution. We identified 132 and 106 selective sweeps associated with domestication and improvement, respectively. Analysis of their tissue‐specific expression patterns indicated that the up‐regulation of selection genes during domestication occurred mostly in fruit and seeds as opposed to other organs. However, during the improvement stage, more up‐regulated selection genes were identified in leaves and seeds than in the other organs. Genome‐wide association studies (GWAS) using 4.24 million single nucleotide polymorphisms (SNPs) revealed 171 loci associated with 26 fruit domestication traits. Among these loci, three candidate genes were highly associated with fruit weight and the sorbitol and catechin content in fruit. We demonstrated that as the allele frequency of the SNPs associated with high polyphenol composition decreased during peach evolution, alleles associated with high sugar content increased significantly. This indicates that there is genetic potential for the breeding of more nutritious fruit with enhanced bioactive polyphenols without disturbing a harmonious sugar and acid balance by crossing with wild species. This study also describes the development of the genomic resources necessary for evolutionary research in peach and provides the large‐scale characterization of key agronomic traits in this crop species.

## Introduction

Populations of existing crop species have been shaped to differing degrees by natural evolutionary forces and anthropogenic influences (Ganopoulos *et al*., 2011). The efficient utilization of diverse genetic resources to improve crop species (Huang and Han, 2014) can be assisted by an understanding of the genetic basis of domestication and improvement. The genetic changes observed in food crop domestication to date have mostly been studied in annual crops that are often propagated from seed each year (Huang *et al*., 2012; Meyer and Purugganan, 2013; Meyer *et al*., 2016; Qi *et al*., 2013). However, perennial fruit crops, which may have lower rates of evolution as they are frequently clonally propagated and have a longer lifespan (Zohary and Spiegel‐Roy, 1975), have not been fully studied with respect to such genetic changes (Meyer and Purugganan, 2013).

Peach (*Prunus persica*) is an important deciduous fruit crop thought to have been domesticated more than 5000 years ago in the middle and lower Changjiang or Pearl River region of China (Cao *et al*., 2016). The subsequent westward movement of peach could have brought it to Persia between the 2nd and 1st century BC. It was then brought to the American continent in two distinct waves (Layne and Bassi, 2008). Due to its palatability, peach is currently widely cultivated in temperate zones, particularly in China, Italy and Spain (FAO, 2014). However, relatively few studies have analysed the evolution of the peach (Akagi *et al*., 2016; Velasco *et al*., 2016) and the genetic variations that have arisen during the peach dispersal process. It remains unclear which candidate genes played significant roles during peach domestication, although comparative genomic studies within 84 peach accessions (including *P. mira*,* P. davidiana*,* P. kansuensis* and landraces) previously identified hundreds of candidate domestication‐related genes (Cao *et al*., 2014).

Genome‐wide association studies (GWAS) are powerful tools for the detection of loci that control phenotypic traits in a range of species (Ogura and Busch, 2015). In peach, signals associated with significant traits were identified using GWAS on data from a 9K SNP array (Micheletti *et al*., 2015). Furthermore, GWAS have been applied to selected domestication traits in peach including fruit weight and soluble solid content (SSC, mainly including acid and sugar) (Cao *et al*., 2016). The exhibition of these traits has been referred to as the ‘domestication syndrome’ in tomato, pepper and melon (Guo and Simmons, 2011). However, the association signals for those traits were not strong due to the small population size utilized in a study on peach (Cao *et al*., 2016). For other important domestication traits, such as the polyphenol content in fruit, using association analysis for gene discovery is also an exciting process, because phenols are a major class of bioactive compounds responsible for health benefits (Sun *et al*., 2002) by reducing damage from oxidative stress (Byrne *et al*., 2009; Cantin *et al*., 2009a) and suppressing the growth and differentiation of human cancer cells (Lea *et al*., 2008).

To further investigate the genomic changes involved in peach domestication and improvement, as well as to identify key genes associated with several domestication traits, such as fruit size, flesh adhesion and texture, and acid, sugar and polyphenol contents in fruit, we performed high‐throughput genome resequencing of 418 peach accessions collected worldwide, including wild related species, wild peach varieties, landraces and improved varieties.

## Results

### Genome variation

**Figure 1:**
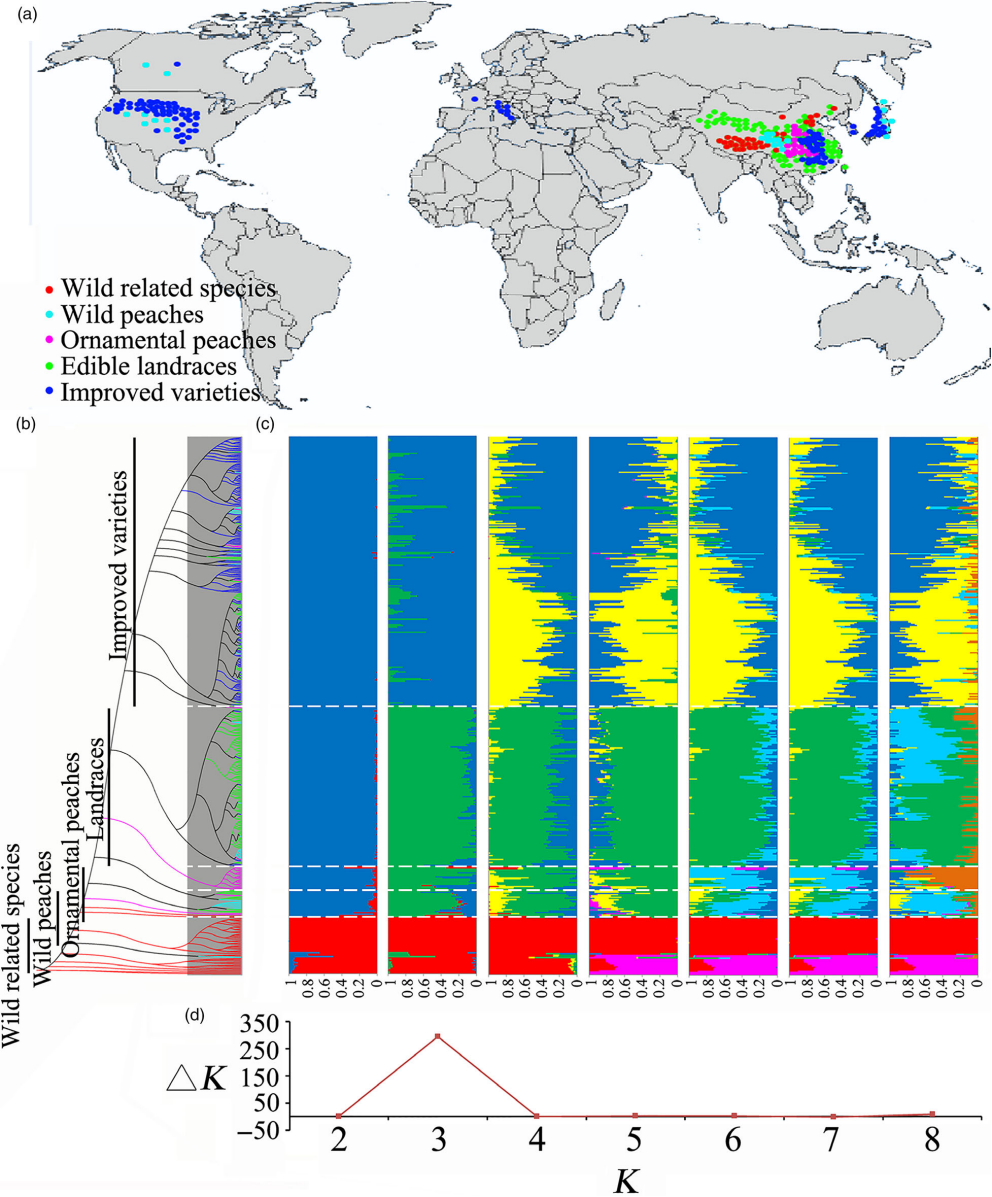
Geographic distribution and genetic structure analysis for 418 peach genotypes. (a) The geographic distribution of the accessions, each of which is represented by a dot on the world map. (b) Phylogenetic tree of 418 accessions calculated from a subset of 46 562 SNPs selected randomly from 2 834 272 common single nucleotide polymorphisms. The five divergent groups, wild related species, wild peach, ornamental peach, landraces and improved varieties, are shown in red, cyan, orange, green and blue, respectively. (c) Population structure of the all peach accessions obtained by STRUCTURE analysis. Different colours represent different populations according to *K* (the number of subpopulations) value analysis. (d) Calculation of Δ*K* to determine the best number of population.

To assess the genetic variations in peach, we previously sampled a core collection of 129 peach accessions distributed throughout the world (Cao *et al*., 2016). For the present study, a total of 418 peach accessions, including 45 varieties belonging to wild related species and 373 belonging to *P. persica*, were resequenced. The former consisted of two *Armeniaca holosericea*, one *P. salicina*, one *P. mongolica*, one *P. tangutica*, and several *P. mira* species that were used as outgroups. The latter included 31 wild peach varieties with small fruit, 29 ornamental lines, 152 edible landraces and 161 improved varieties collected from different countries around the world. The geographic distributions of the accessions included in this report included China, Korea, Japan, Italy, Canada and the United States (Figure 1a and Table S1).

Approximately 1.17 Tb of clean sequencing reads was generated from the 418 peach accessions, with an average depth of 7.33× and a coverage of 93.96% (Table S1). We identified a total of 4 224 349 single nucleotide polymorphisms (SNPs) among the accessions. Among those, the number of SNPs in the improved varieties (1 921 556) was lower than that in the wild related species (3 621 218), wild peaches (3 696 694) and edible landraces (3 038 161), and higher than that in ornamental peaches (1 233 778). Additionally, 41 582 SNPs were accession‐specific (Figure S1), and wild related species had more accession‐specific SNPs (26 246) than the improved varieties (6065), edible landraces (5229), ornamental peaches (2402) and wild peaches (1640) (Table S2). Of the 2 830 238 common SNPs, which had an average density of 79.74 SNPs per kilobase (Table S3), 492 507 were found in exons, including 2480 putatively damaging SNPs (stop codon gain/loss) in 1965 genes. The validation of 22 randomly selected SNPs on the Sequenom MassARRAY demonstrated a 95.3% accuracy for the identification of SNPs (Data S1 and Table S4).

### Population structure of worldwide peach germplasm

On the basis of phylogenetic (Figure 1b) and population structure analyses (Figure 1c), all wild lines were classified into one clade when *K* = 2. These results support the hypothesis that all currently grown domesticated and improved peaches originated from a single domestication event (Cao *et al*., 2014). However, *P. tangutica* and *P. mongolica* showed a close relationship with *P. davidiana* (Figure S2). The results may explain early writings that classified almond and peach under the same species, but these were later divided into two groups, although they possibly shared the same putative ancestor, as the mesocarp of the almond becomes dry and splits at maturity (Layne and Bassi, 2008). The other domesticated accessions were further grouped into two main clades when *K* = 3. One clade included most of the landraces, and the other contained mainly the improved varieties. To analyse the dispersal of peach varieties worldwide, the improved variety subgroup was also enlarged in the population structure (Figure S3a). We found that the population was divided into two subclades. The first subclade contained varieties mainly developed from the ‘Chinese Cling’ peach by researchers in China, Japan and South Korea. The second subclade included varieties from Europe and America. When the latter accessions were compared, the distribution of improved varieties in China was found to be random, indicating a high degree of polymorphism (Figure S3b). The wild and ornamental peaches did not separate from the landraces until *K* = 6. The wild and ornamental peaches are closely genetically related, as they were not discrete until *K* = 8. Structure analyses (Figure 1c) showed that the optimal number of subpopulations for the 418 accessions was *K* = 3 after calculation of the most likely number of clusters, Δ(*K*) (Figure 1d).

The genetic diversity (π), which was calculated in 50‐kb windows across the peach genome based on the SNP data, was estimated at a mean value of ~2.10 × 10^−3^ for the wild related species population. The value decreased obviously among wild and ornamental peach varieties (~1.14 × 10^−3^) and landraces (~1.07 × 10^−3^) to ~0.89 × 10^−3^ in improved varieties. The results suggested that approximately half of its genetic diversity has been lost during peach domestication from wild species to landraces. However, most genetic diversity was retained during the improvement of landraces to improved varieties. The result agreed with the fixation index value (*F*_ST_) between the landraces and improved varieties (0.06, Table S5). Meanwhile, we detected polymorphism differences among improved varieties originating from different regions (Figure S4). A highly similar π value across the genome was noted between peaches from China, Europe and America (*r* = 0.982), followed by that between peaches from China, Japan and South Korea (*r* = 0.951). These results suggested a low level of genetic differentiation among different improved varieties.

### Selection signals during evolution

**Figure 2:**
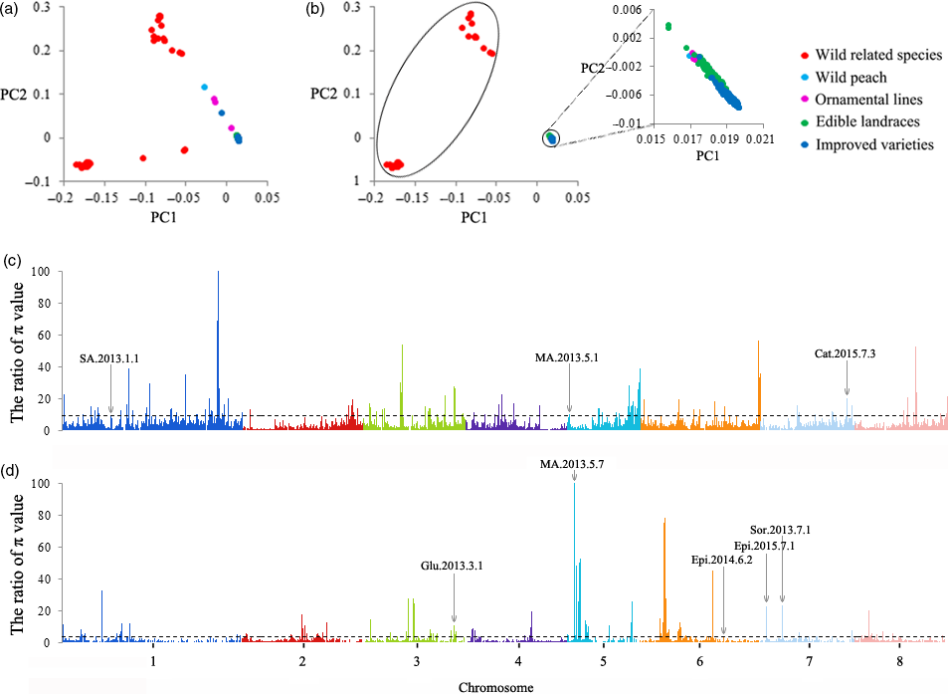
Whole‐genome screening of the selective sweeps that occurred during peach domestication and improvement. (a) Principal component analysis of all 418 peach accessions, including wild related species, wild and ornamental peach, edible landraces and improved peach varieties considered in the present study. All common SNPs identified were used in the analysis. Each peach accession is represented by a point in the two‐dimensional space defined by the eigenvectors of the first and second principal components. Different colours represent different peach subpopulations. (b) Principal component analysis of the 323 peach accessions filtered according to the STRUCTURE analysis. (c) Whole‐genome screen of the selective sweeps that occurred during peach domestication. The ratios of π value between wild related species and varieties belonging to *Prunus persica* (wild peach, ornamental peach, edible landraces and improved varieties) calculated for the eight chromosomes are plotted against position on each of the chromosomes. The black dotted line indicates the genome‐wide threshold of selection signals (9.06). The locus name is shown in Table S11. (d) Whole‐genome screen of the selective sweeps that occurred during peach improvement. The black dotted line indicates the genome‐wide threshold of the signal (π_edible landraces_/π_improved varieties_ ≥ 3.84). The locus name is shown in Table S11.

To detect potential selective signals during peach domestication (wild related species versus landraces and improved varieties) and improvement (landraces versus improved varieties), we screened the selection sweeps through the comparison of whole‐genome polymorphism data between different populations. An initial principal component analysis (PCA) of whole‐genome polymorphism data from all 418 accessions displayed a continuous distribution (Figure 2a). When accessions exhibiting admixture in the STRUCTURE analysis were removed, a PCA based on 2 785 339 SNPs in 323 accessions illustrated 3 distinct clusters (Figure 2b) comprising 2 distinctively separated groups of ‘wild related’ species and a compact group comprising wild varieties, ornamental varieties, edible landraces and improved varieties of peach. The ratios of the π between wild related species and that between all varieties of *P. persica* calculated along the genome were plotted (Figure 2c) and revealed 132 probable selective sweeps (Table S6) encompassing 1212 domestication genes. An analogous plot of the ratio of the π values between edible landraces and improved varieties identified 108 probable selective sweeps (Figure 2d and Table S6) with 869 improvement genes. The selection signals were modest, and the average π_landrace_/π_improved variety_ value was calculated as 2.40, which was lower than that of π_wild related species_/π_total *P. persica*_ (3.89), suggesting that selection was weaker during modern genetic improvement than during domestication (Fang *et al*., 2017). The domestication‐related regions in the peach genome were mainly found within Chr. 1, 3 and 5, while the improvement‐related regions were distributed over Chr. 1, 2, 3, 5 and 6 (Figure S5). In addition, the cross‐population extended haplotype homozygosity (XP‐EHH) test (Sabeti *et al*., 2007) revealed additional selection sweeps that were not identified by the π‐based approach. Forty of the 164 domestication‐related sweeps and 61 of the 165 improvement‐related sweeps that were detected according to π value were confirmed in the XP‐EHH analysis (Table S6).

Taking an important domesticated trait of peach, fruit weight, as an example, we found that of the 45 reported QTLs for this trait, only 12 overlapped with selection sweeps during domestication and improvement (Table S7). We found that previously uncharacterized sweeps were smaller than the QTLs located in unsaturated linkage maps using restriction fragment length polymorphism or simple sequence repeat markers. These defined regions will be helpful for the identification of genes that govern domestication‐related traits (Zhou *et al*., 2015). For instance, within or near the above selection regions overlapping with the QTLs for fruit weight (Table S7), several genes encoding Cell Number Regulator protein (*Prupe.2G276700*,* Prupe.5G219300* and *Prupe.5G219400*) and cytochrome P450 78A (*Prupe.8G046400*) were significant.

**Figure 3:**
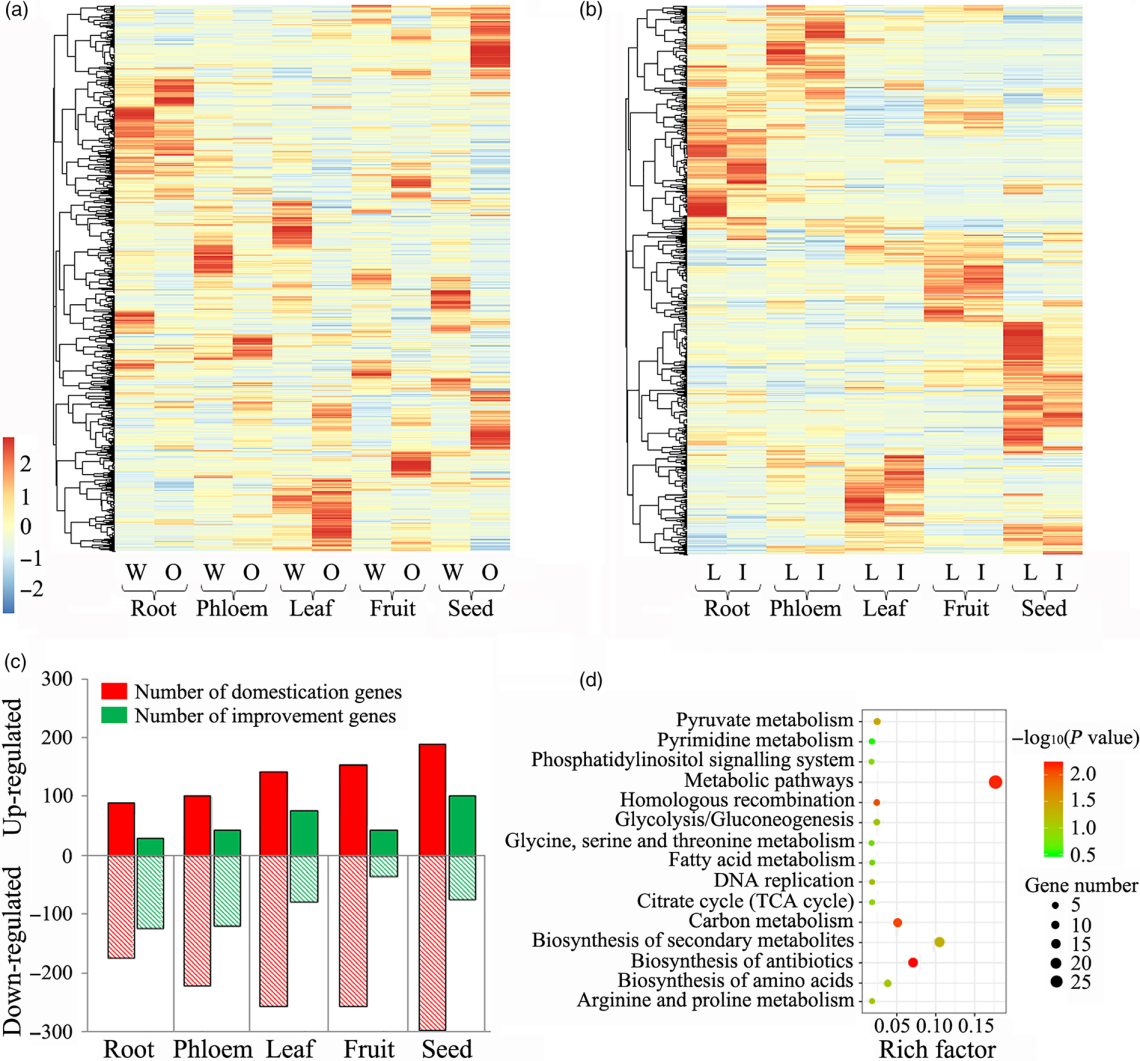
Tissue‐specific expression of genes from four groups of peaches. (a) The expression of genes associated with speciation in five tissues of wild related species (W) and ornamental peach (O). (b) The expression of genes associated with improvement in five tissues of landraces (L) and improved varieties (I). (c) The proportions of differentially expressed genes associated with domestication and improvement in five different tissues of peach. (d) The Kyoto Encyclopedia of Genes and Genomes (KEGG) pathway analysis for the domestication genes which exhibited up‐regulated expression in fruit.

We further investigated the tissue‐specific expression of the genes identified in selection sweeps (Figure 3a,b) and classified them into as unregulated or down‐regulated genes during peach domestication and improvement. We found that more up‐regulated domestication‐related genes were identified using the π‐based approaches in seeds, fruit and leaves than in phloem and roots. However, more up‐regulated improvement‐related genes were identified in seeds and leaves than in other tissues (Figure 3c). Therefore, seeds were not the focus of the following analysis, because most of the expressed genes in this type of tissue were persistently up‐regulated during peach evolution. Then, the Kyoto Encyclopedia of Genes and Genomes (KEGG) pathways of the up‐regulated domestication‐related genes in the fruit were emphasis‐analysed, because most of them were not up‐regulated during species improvement. The KEGG enrichment analysis indicated that genes involved in the biosynthesis of secondary metabolites, antibiotics, carbohydrates and amino acids were very important for the domestication of peach (Figure 3d). The most highly up‐regulated domestication‐related gene was *Prupe.2G263600* (Chr. 2: 27 148 381–27 150 400 bp), which encodes an expansin A8 and is close to a previously identified SSR marker (UDP98‐406, Chr. 2: 28 001 334–28 001 432 bp) for fruit weight (Eduardo *et al*., 2011). The other up‐regulated domestication‐related genes included *Prupe.6G362400*, which encodes an aspartic proteinase‐like protein; *Prupe.2G135500*, which encodes a resistance protein; and *Prupe.5G233100*, which encodes an auxin efflux carrier.

The candidate genes in the sweep regions with an obvious selection signal (Table S8) and those in the large‐effect sweep regions (Table S9) have been presented in Appendix S1.

### Genome‐wide association analysis of several domestication traits

A clear population structure analysis helped us to perform accurate GWAS to better identify true domestication‐ or improvement‐related genes from the above selection sweeps. In this study, we performed GWAS for several domestication‐related traits of fruit in peach. The phenotypes can be classified into two categories: fruit/stone size and fruit interior quality. To prevent the generation of spurious associations resulting from population stratification, we did not measure all agronomic traits in all populations; we only used phenotypes obtained from the 313 filtered accessions including edible landraces and improved varieties (Figure S6 and Table S10). In addition, we indeed found that population structure was a key cause of spurious association (Figure S7). Using a linear mixed model with 988 306 filtered SNPs in the resulting population and accounting for Q matrix and kinship values, we identified 9, 21, 11, 9, 23, 28, 22 and 48 association signals for the traits of fruit size stone size, fruit/stone weight, flesh adhesion/texture, SSC/titratable acidity (TA), and acid, sugar and polyphenol contents, respectively (Figures S8–S23 and Table S11). Among them, 27 association signals for the same location were shared among at least two different traits, including one locus associated with malic acid content, fructose content, chlorogenic acid content and fruit weight (Chr. 2: 6.59–6.89 Mb); four loci associated with catechin and epicatechin content (Chr. 2: 13.74 Mb; Chr. 3: 21.02 Mb; Chr. 5: 10.67 Mb; and Chr. 7: 9.76–9.82 Mb); and three loci associated with fruit vertical diameter and stone length (Chr. 6: 26.88–26.92 Mb; Chr. 6: 28.47–28.50 Mb; and Chr. 6: 30.39 Mb). The co‐location of these QTLs is supported by the correlations between these domestication traits (Figure S24).

Flesh adhesion and texture are two important domestication traits in peach. In the present study, we found that the percentage of clingstone and non‐melting peach accessions decreased with evolution. An obvious difference was found among the improved varieties in the subpopulations of China, Japan and South Korea, Europe and America (Figure S25), reflecting complex breeding efforts in different countries. We detected three clear signals associated with flesh adhesion and one clear signal associated with texture (Figure S11 and Table S11). The peak signals for the above traits were only 8 and 800 kb away, respectively, from the two polygalacturonase genes previously reported to regulate flesh adhesion and texture (Gu *et al*., 2016).

Fruit weight (FW) and SSC were focused on because they were determined to be important traits in previous crop production and domestication studies of horticultural species (Sun *et al*., 2017). We identified a clear one year‐stable signal associated with fruit weight on Chr. 6, although the association signal in 2016 was lower than the genome‐wide threshold (Figure S12). The signal was also found to overlap in GWAS of fruit vertical diameter, fruit cheek diameter, fruit suture diameter and stone length (Figures S8 and S9a). These results indicated that the genetic control of this complex trait may be related to the expansion and division of mesocarp cells. However, GWAS of the total SSC yielded no clear association signal (Figure S13). This result is consistent with the significant differences in the fruit weight (FW) of domesticated peach (edible landraces and improved varieties) versus wild accessions over all 418 accessions, whereas there were no obvious differences in the SSC among accessions (Figure S26). The SSC is a complex trait that includes sugars (fructose, glucose, sorbitol and sucrose), acids (citric acid, malic acid, quininic acid and succinic acid), vitamins, minerals and other water‐soluble compounds. Malic acid (56.67% ± 5.51) and sucrose (68.78% ± 7.23) were the major acid and sugar components in the fruit, respectively (Table S10 and Figure S27), and loci were identified on Chr. 2 and 5 for malic acid content. Although no association was identified for fruit sucrose content, two association signals for fructose in Chr. 2 and 3; seven association signals for glucose in Chr. 1, 3 and 8; and thirteen association signals for sorbitol in Chr. 1, 2, 4, 5, 6 and 7 were located (Table S11). In peach, the sugar/acid ratio is a major organoleptic quality trait of the mature fruit (Dantec *et al*., 2010). Therefore, we further identified two association regions with a moderate effect on the sugar/acid in Chr. 5 (21 QTLs) and one region with a minor effect on the sugar/acid ratio in Chr. 2 (2 QTLs). Most of them coincided with the positions of QTLs for malic acid content.

As increased fruit size and enhanced flavour are the two most important outcomes of the domestication of fruit crops (Meyer and Purugganan, 2013), we expected genes directly involved in the control of these traits to have been under strong selection (Akagi *et al*., 2016). However, in general, of the 171 signals associated with fruit/stone size, fruit weight, sugar and acid content identified by GWAS (Table S11), only thirteen and fifteen were under selection, as detected by their π values and XP‐EHH analysis, respectively (Figures 2c,d and S28a,b). Among the association signals for fruit size, only one QTL (SL.2014.6.9) was found to be selected. It may be that peach shape appears is stable and round regardless of the level of domestication, but stone shape appears oval and varies from one variety to another (Quilot *et al*., 2004). In addition, no overlap was detected between the association signals for flesh adhesion and texture and the selective sweeps. The results were consistent with our findings on the tissue‐specific expression of selection genes, in which fewer improvement‐related genes were regulated in fruit tissue than in leaves and seeds (Figure 3c). Taken together, our results indicate that fruit cannot be thought of as a primary selection organ during improvement but can during domestication.

Next, we focused only on the allele frequency of each associated SNP for each trait (Figure S29) instead of screening the selection sweeps by analysing genetic polymorphism at the genomic level. We demonstrated that the allelotype of SNPs associated with polyphenol content changed noticeably (with minor allele frequency dropping sharply) during evolution from wild species to improved varieties, and trended towards heavy homozygosity for polyphenol. However, the allelotype of the SNPs at loci associated with fruit size, flesh adhesion/texture and acid content showed a trend in the opposite direction towards heterozygosity. This explains why the QTLs of these latter traits were not under selection during evolution.

### In‐depth analyses of loci associated with principal fruit quality traits

#### Fruit weight

**Figure 4:**
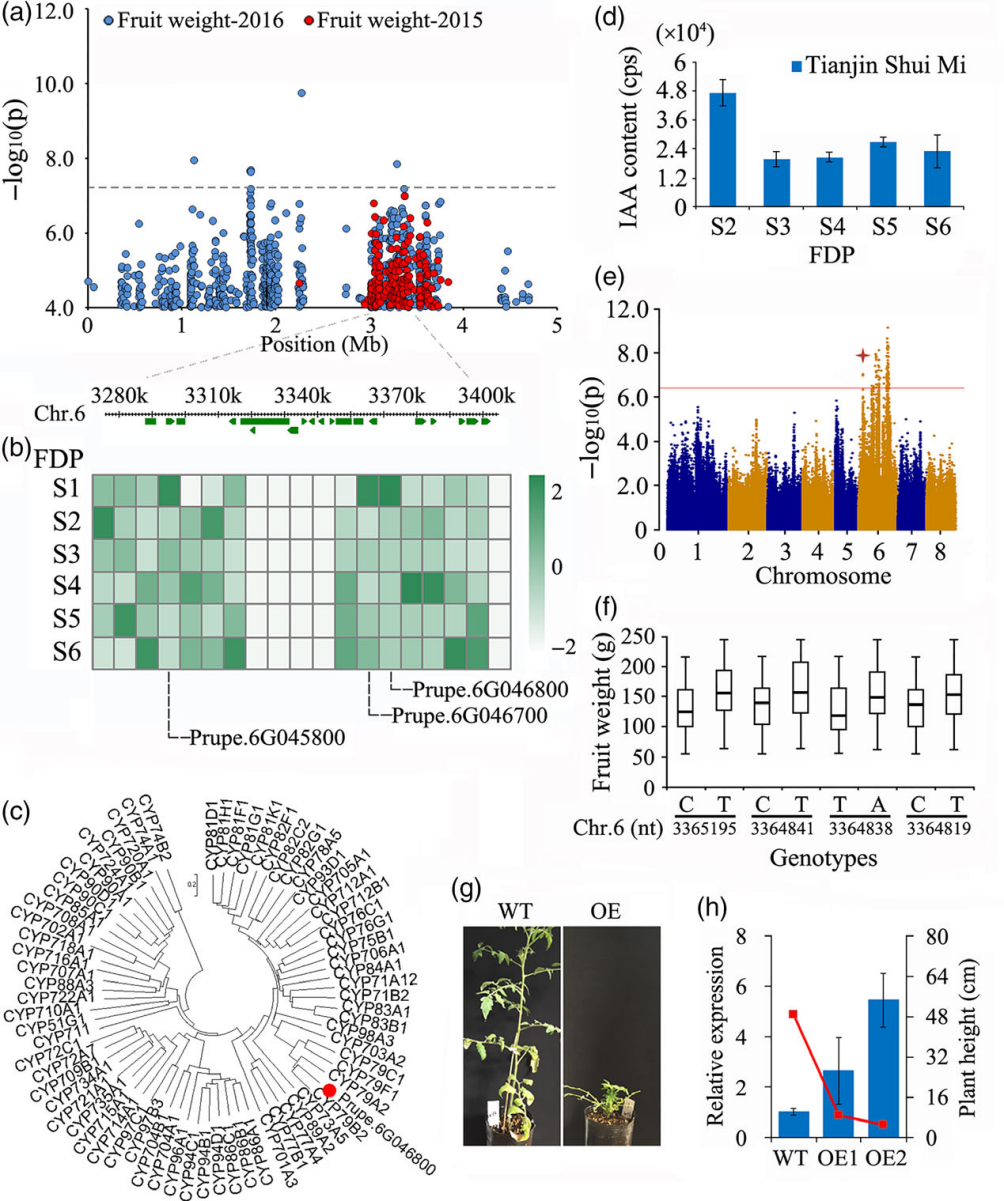
Association results for fruit weight and the expression profiles of candidate genes in peach. (a) SNP loci associated with fruit weight in chromosome 6. Genomic position (*x*‐axis) is plotted against its significance expressed as ‐log_10_ *P* value (*y*‐axis). The black dotted horizontal line indicates the genome‐wide significance threshold (5.06 × 10^−8^). The genomic position indicated by a black dashed vertical line spans approximately 50 kb on either side of the overlapped peak association SNPs of fruit weight in 2015 and 2016. The annotated candidate genes are represented by green boxes along the chromosome. (b) Expression of 14 candidate genes in the regions of overlapped association signal in 2015 and 2016 in ‘Tianjin Shui Mi’ peach, obtained with RNA‐Seq, during the six stages of fruit development period (FDP) in 2015. S1–S6 corresponds to 30, 45, 60, 75, 90 and 105 days after full bloom. (c) Phylogenetic analysis of *Prupe.6G046800* and other *cytochrome P450* (*CYP*) genes in *Arabidopsis thaliana*. (d) The IAA concentration in fruit of ‘Tianjin Shui Mi’ peach at different times after full bloom. (e) The genome‐wide association study of IAA concentration in mature fruit. The asterisk indicates the position of the association signal for fruit weight on chromosome 6. (f) Allelotypes of *Prupe.6G046800* identified using 75 accessions and their relationship with fruit weight. In the bar plots, the highest and lowest quartiles are displayed above and below the box and the line in each box represents the mean value. (g) Overexpression (OE) of *Prupe.6G046800* in tomato led to low plant height compared with wild type (WT). (h) Expression of *Prupe.6G046800* in the transgenic and control tomato was tested with qRT‐PCR. Tomato *actin* (GenBank: FJ532351) was used as an internal control. Data are represented as average values with SD (*n* = 3 technical replicates).

A locus close to 3 Mb on Chr. 6 was significantly associated with FW in peach samples collected in 2015 and 2016 (Figure S12 and Table S11). Potential candidate genes located within a 50‐kbp window spanning the locus (Figure 4a) are listed in Table S12. Examination of the expression pattern of genes during the fruit development period (FDP) (Figure 4b and Table S13) identified three genes (*Prupe.6G045800*,* Prupe.6G046700* and *Prupe.6G046800*, which encode an endonuclease, a ribonucleoprotein and isoleucine N‐monooxygenase, respectively). These exhibited peak expression at the initial phases of the FDP (Figure 4b), which corresponds to the cell division stage. Phylogenetic analysis of *Prupe.6G046800* showed that it belongs to the *Cytochrome P450* (*CYP*) *79B* subfamily (Figure 4c) and shows moderate similarity (54%) with its homologue in *Arabidopsis*. Moreover, we detected high concentrations of IAA in fruit at the early stage of development (Figure 4d) coinciding with the expression of *Prupe.6G046800* (Figure 4b), whose homologue in *Arabidopsis* is involved in IAA biosynthesis (Mikkelsen *et al*., 2000). Furthermore, an association signal for the IAA concentration in mature fruit was detected at a similar position in this study (Figure 4e). Since the candidate gene is located distant from the SNP at the peak of QTL FW.2016.6.3, we cloned and sequenced the promoter and coding region of the gene. Using a subset of 75 varieties (Table S14), we identified four mutations in the promoter region of *Prupe.6G046800* strongly associated with fruit weight (Figure 4f). To confirm the effect of this gene on fruit weight, we further overexpressed the gene in tomato. The transgenic tomato plants were shorter than the vector control plants (Figure 4g,h), which is consistent with the results in *Arabidopsis* (Mikkelsen *et al*., 2000). However, we did not obtain mature fruit from transgenic tomato because the growth of the plant was severely reduced. Therefore, we inferred that this previously undescribed gene underlies fruit development in peach.

#### Sorbitol content

**Figure 5:**
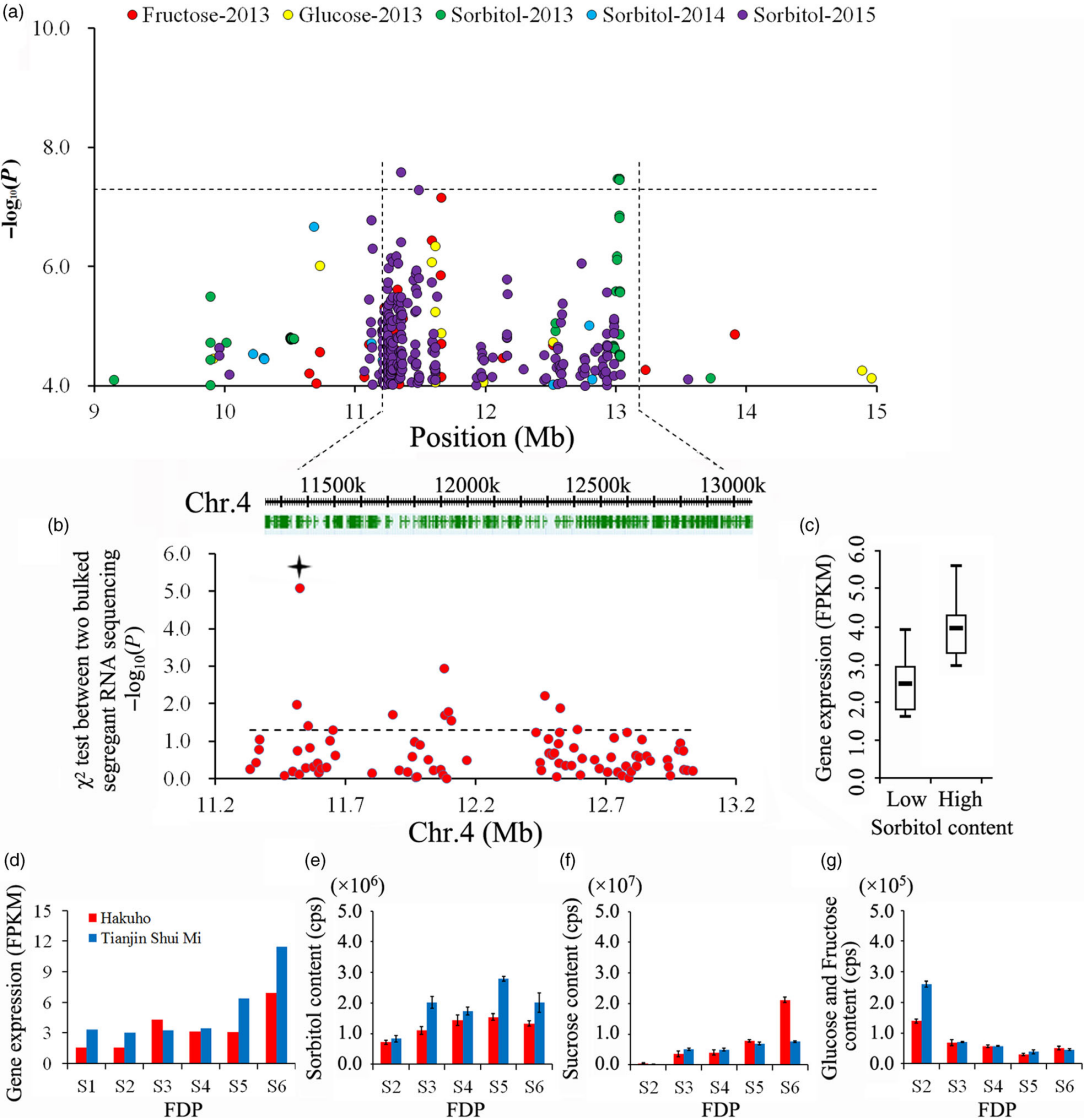
Association results for fruit sugar content and the expression profiles of candidate genes for control of sugar content in peach. (a) Identification of the position of loci on chromosome 4 associated with fructose, glucose and sorbitol content. Genomic position (*x*‐axis) is plotted against its significance expressed as the ‐log_10_ *P* value (*y*‐axis). The black dotted horizontal line indicates the genome‐wide significance threshold (5.06 × 10^−8^). The genomic position indicated spans approximately 50 Kb on either side of the two SNPs associated with sorbitol content in 2013 and 2015, indicated by black dashed vertical lines. The annotated candidate genes are represented by the green boxes displayed below the plot. (b) Bulked segregant RNA‐Seq analysis of 180 selected candidate genes in pool of mature fruit of 15 low sorbitol content varieties and 15 high sorbitol varieties. The position of *Prupe.4G191900* which showed the obvious differential expression between two bulks is indicated with a star. (c) Expression of *Prupe.4G191900* in the two bulked segregant pools. (d) Relative expression of *Prpue.4G191900*, obtained by RNA‐Seq in two varieties with differing fruit sugar content, during six stages (S1–S6) of the fruit developmental period (FDP). (e) Changes in sorbitol content in two varieties during five developmental periods (S2–S6), measured by high‐throughput liquid chromatogram (LC)–mass spectrometry (MS)/MS. (f) Change in sucrose content in two varieties during five stages (S2–S6) of FDP, measured by UPLC‐MS. (g) Change in glucose and fructose content in fruit of two varieties during five stages (S2–S6) of FDP, measured by LC‐MS/MS. Values for each sample represent mean ± SD for triplicates phenotyped.

We identified 180 genes distributed in a 50‐kbp region of Chr. 4 spanning the region containing the two SNPs most highly associated with year‐stable sorbitol QTLs (Figure 5a). RNA‐Seq expression of a bulked pool of mature fruit from 15 varieties with low and high levels of sorbitol (Table S15) identified a total of 85 genes with expression levels (fragments per kb per million reads, FPKM) ≥ 1.0 (Table S16). Of these, 10 genes exhibited noticeably differential expression between the two bulks according to the chi‐square test (Figure 5b). *Prupe.4G191900*, which exhibited the lowest *P* value, was chosen for comparative analysis of gene expression in ‘Hakuho’ and ‘Tianjin Shui Mi’ peaches (Figure 5c). This gene is also close to the peak association signal for fructose and glucose content in peach samples collected in 2013 (Figure 5a). When we investigated the expression of this gene during development in two varieties with differing fruit sugar content, the gene expression was detected at each stage of fruit development, with the highest value measured at stage S6 (fruit ripening). In the last stage, higher gene expression was observed in the ‘Tianjin Shui Mi’ peach than in the ‘Hakuho’ peach (Figure 5d). The content of one sugar, sorbitol, increased with fruit development and was present in higher quantities in the ‘Tianjin Shui Mi’ peach than in the ‘Hakuho’ peach (Figure 5e), but this was not observed for sucrose and glucose (Figure 5f,g).

#### Polyphenols

**Figure 6:**
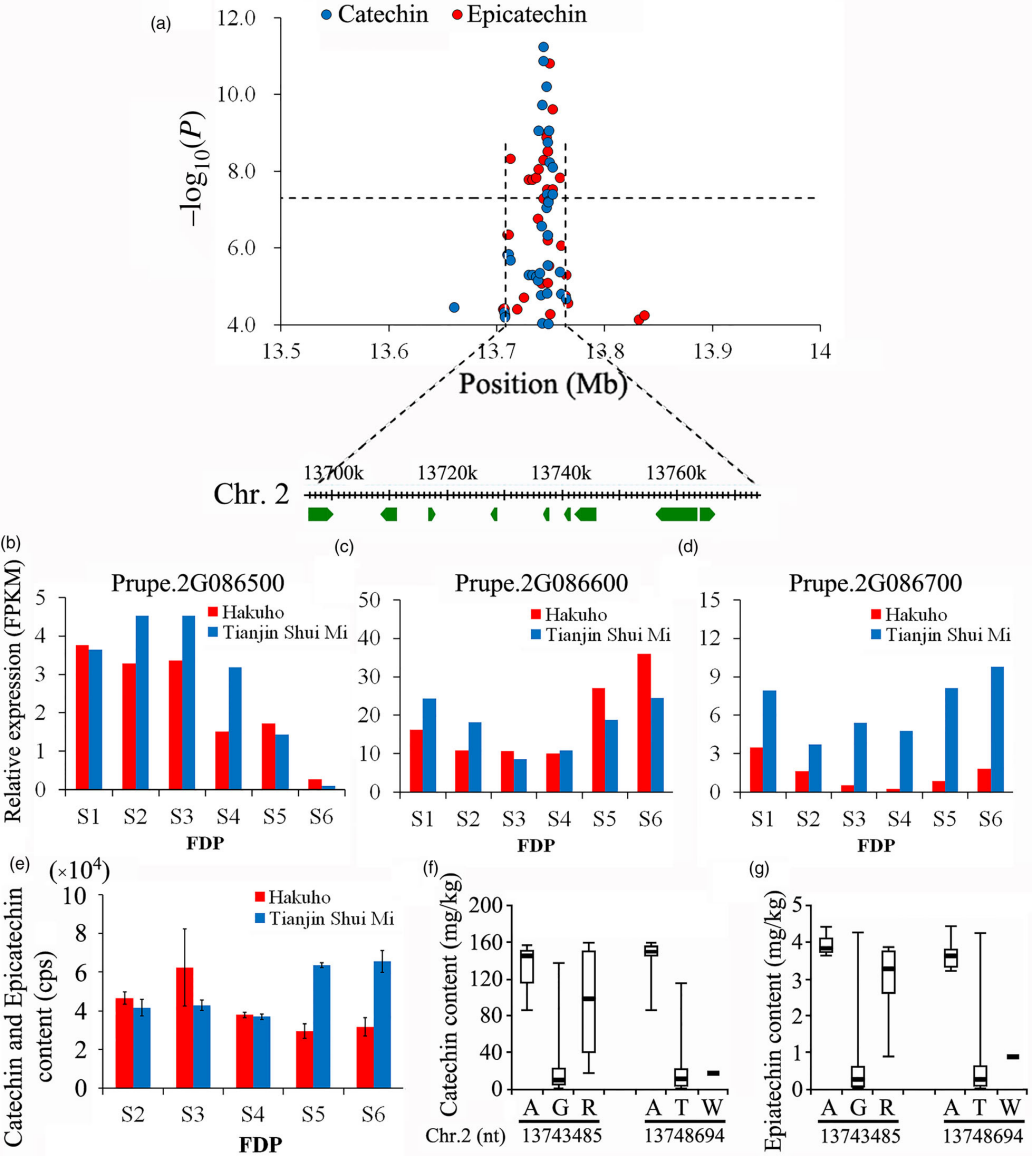
Association results for overlapping loci for catechin and epicatechin content in peach fruit and the expression of candidate genes during fruit development. (a) Position of the locus associated with catechin and epicatechin content in fruit on chromosome 6. Genomic position (*x*‐axis) is plotted against its significance expressed as −log_10_ *P* value (*y*‐axis). The black dotted horizontal line indicates the genome‐wide significance threshold (5.06 × 10^−8^). The genomic position depicted spans approximately 50 Kb on either side of the two SNPs associated with catechin content in fruit in 2015, indicated by a black dashed vertical line. The annotated candidate genes are represented by the green boxes displayed below the plot. (b) Expression of the candidate gene *Prupe.2G086500* in ‘Hakuho’ and ‘Tianjinshuimi’, obtained with RNA‐Seq, during six stages of the fruit developmental period (FDP) from 24 April (S1) to 8 July (S6). The red and blue colour bars represent ‘Hakuho’ and ‘Tianjinshuimi’, respectively. The labels are the same in Fig. 4c–e following. (c) Expression of the candidate gene *Prupe.2G086600* in ‘Hakuho’ and ‘Tianjinshuimi’. (d) Expression of the candidate gene *Prupe.2G087000* in ‘Hakuho’ and ‘Tianjinshuimi’. (e) Change in catechin and epicatechin content in fruit of ‘Hakuho’ and ‘Tianjinshuimi’ during five stages (S2–S6) of FDP, measured by LC‐MS/MS. Values for each sample represent mean ± SD for triplicates phenotyped. (f) Genotypes of *Prupe.2G087000* identified using resequencing data from 313 accessions and their allelotypic relationship with fruit catechin content measured by ultra‐high‐performance liquid chromatography (UPLC)–mass spectrometry (MS). In the bar plots, the highest and lowest quartiles were displayed above and below the box and the line in each box represents the mean. (g) Genotypes of *Prupe.2G087000* identified from resequencing data of 313 accessions together with their allelotypic relationship with epicatechin content in fruit measured by UPLC‐MS.

Forty‐eight association signals for five polyphenol compounds were found (Table S11), with the majority distributed over Chr. 1, 2 and 3. We identified four loci where single‐year QTLs controlling catechin and epicatechin content overlapped (Table S11). Focusing on the overlapping QTLs with the highest *P* value located from 13 721 089 bp to 13 771 089 bp on Chr. 2 (Figure 6a), the expression of 9 genes was analysed. Among them, three genes, *Prupe.2G086500*,* Prupe.2G086600*, and *Prupe.2G087000*, exhibited high expression levels (FPKM ≥ 1) during the fruit development period (Figure 6b‐d). The expression of *Prupe.2G086600* and *Prupe.2G087000* also followed a similar pattern of increased expression during fruit ripening. Of the two genes, *Prupe.2G087000* was more differentially expressed between the two peach varieties, ‘Hakuho’ and ‘Tianjin Shui Mi’. The catechin and epicatechin content remained constant in the two varieties at the early stage of fruit development. However, the levels of these polyphenols tended to increase as the fruit from the ‘Tianjin Shui Mi’ peach ripened (Figure 6e). The increased expression level of *Prupe.2G086600* did not parallel the constant change in catechin and epicatechin content in the ‘Hakuho’ variety. *Prupe.2G087000* was located close to the SNP at the peak of QTL Cat.2015.2.1 and overlapped with a SNP at the Epi.2015.2.1 peak. Allelotype analysis of these SNPs in all 313 varieties confirmed that *Prupe.2G087000* was significantly related to the catechin (Figure 6f) and epicatechin (Figure 6g) content in mature fruit.

## Discussion

In this study, we developed a clear understanding of the molecular basis of the evolution of *P. persica* from a wild ancestral related species, *P. mira*, distributed in the harsh climate of the Qinghai–Tibet Plateau to the improved varieties that are globally distributed. We expanded on our previous study (Cao *et al*., 2014), employing a more detailed classification of a larger number of accessions. We used GWAS to identify loci associated with fruit/stone size, flesh adhesion or texture, and acid, sugar and polyphenol contents in fruit that might be involved in the evolution of *P. persica* and the RNA profiling of candidate selection genes across different tissues to identify highly differentially expressed genes (DEGs) that may be related to the evolutionary processes of domestication and improvement.

### Fruit weight

Previous studies reported that the FW of peach is controlled by multiple QTLs located from Chr. 1 to 8 (Abbott *et al*., 1998; Desnoues *et al*., 2016; Dirlewanger *et al*., 1999; Eduardo *et al*., 2011; Etienne *et al*., 2002; Linge *et al*., 2015; Quilot *et al*., 2004; Zeballos *et al*., 2016). However, the key loci had not been determined to date, preventing the identification of the responsible candidate gene. Using GWAS, we identified a locus near the top of Chr. 6 close to 3 Mb that was significantly associated with fruit weight (Figure 4) and contained the candidate gene *Prupe.6G046800*. Previously reported QTLs for the control of FW on Chr. 6 were at the bottom of the chromosome (Desnoues *et al*., 2016; Dirlewanger *et al*., 1999; Eduardo *et al*., 2011), distant from the position of the QTL we identified. *Prupe.6G046800* is a cytochrome P450 gene that is highly expressed during the initial stage of the FDP that coincides with cell division. This timing is significant, as plant organ size is more affected by cell number than by cell size (Guo and Simmons, 2011; Scorza *et al*., 1991; Yamaguchi *et al*., 2002). In addition, we identified four mutations strongly associated with fruit weight in the promoter region of *Prupe.6G046800*. Cytochrome P450s are involved in a wide range of biosynthetic reactions in plants that lead to the production of plant hormones, fatty acid conjugates, secondary metabolites and lignins (Schuler and Werck‐Reichhart, 2003). The homologous genes in *Arabidopsis* (*CYP79B2* and *CYP79B3*) convert tryptophan to indole‐3‐acetaldoxime (IAOx), a precursor to indole‐3‐acetic acid (IAA) and indole glucosinolates (Hull *et al*., 2000). IAA is a major naturally occurring auxin with a well‐documented role in plant development (Davies, 2010), and the concentration of IAA was elevated at the same period of the FDP when *Prupe.6G046800* was highly expressed. The homologous gene in tomato, *SlKLUH* (*CYP78A* subfamily), has been reported to be the causal gene of the tomato FW2.3 QTL as well as associated with fruit mass (Chakrabarti *et al*., 2013). Moreover, *PaCYP78A9,* a cytochrome P450 gene in cherry, affected cherry fruit size by mediating mesocarp cell proliferation and expansion during fruit growth and development (Qi *et al*., 2017). Consequently, we believe that *Prupe.6G046800* is a valid candidate gene for the control of peach fruit weight.

### Sugars and acids

Sugar content and composition are important factors in determining the quality of peaches. In ripe peaches, the main soluble sugars are sucrose, fructose and glucose, with sorbitol detected at low levels (Moing *et al*., 1998). In this study, we, like other researchers (Chakrabarti *et al*., 2013), found it difficult to identify QTLs for fruit sugar content, with year‐unstable QTLs characterized by low log odds (LOD) scores and small percentages of explained phenotypic variability. In our GWAS, we identified 21 year‐unstable loci related to sugar content over three successive years, with a single locus associated with sorbitol detected over two successive years (Figure 5). This result agreed with the previous finding that both fructose and sorbitol contents are less influenced by the prevailing environmental conditions over the growing season than other sugars (Cantin *et al*., 2009b). This association signal for sorbitol was close to an important QTL previously reported for the control of fructose, glucose and sorbitol contents, the total sugar content and the SSC (Abbott *et al*., 1998; Cirilli *et al*., 2016; Salazar *et al*., 2014). Our results indicated that the control of complex domestication traits in peach may have resulted from a combination of minor effect QTLs that are unstable across years, as reported for perennial fruit crops in general by Miller and Gross (2011). Although we identified 180 genes within the 50‐kbp region between the SNPs with the highest association with fruit sorbitol content (11 352 337–13 028 258 bp on Chr. 4) (Figure 5a), these were reduced to one candidate gene by expression analysis of bulked mature fruit from 15 varieties with low and high sorbitol content (Figure 5b). This strategy enabled us to select the diacylglycerol kinase encoding gene on Chr. 4, *Prupe.4G191900*, as a candidate gene for the year‐stable control of sorbitol content. Although the position of this gene is also close to the position of the peak association signals for fructose and glucose content, expression studies did not support a role for *Prupe*.*4G191900* in the control of the content of these sugars in fruit. As sorbitol is the main translocated carbon in peach, the gene identified in the study can serve as a powerful tool for dissecting sorbitol metabolism and the regulation of sugar levels in fruit.

As fruit acids have a major influence on flavour perception (Malundo *et al*., 2001), a major goal of this study was to investigate the control of the predominant acid in peach fruit, malic acid. The region associated with malic acid content at the top of Chr. 5 identified in our GWAS analysis is consistent with the results of previous studies using map‐based cloning (Boudehri *et al*., 2009) and the evaluation of malic acid as a qualitative trait in non‐acidic fruit (Cao *et al*., 2016). In addition, a total of 16 genes were found at the QTLs controlling malic acid content from 5 357 897 bp to 5 571 577 bp in Chr. 2. Six genes are involved in gene transcription regulation and protein function, and two genes are responsible for controlling receptor‐like protein kinase.

### Health‐related compounds

The health‐related compound content is a major consideration when consumers purchase fruit, in addition to flavour, aroma and visual appearance (Battino *et al*., 2009). Polyphenolic compounds, especially flavonoids, convey health benefits due to their ability to scavenge radicals (Yao *et al*., 2004). Based on the genes underlying the overlapping single‐year loci on Chr. 2 for the control of fruit catechin and epicatechin content identified in GWAS, we identified a candidate gene, *Prupe. 2G087000*, that encodes ribosomal protein (Figure 6). This gene exhibited a high expression level during fruit development and was differentially expressed between two peach varieties that exhibited changes in the combined content of catechin and epicatechin as their fruit developed towards maturity. Furthermore, analysis of the SNPs associated with the QTLs for catechin and epicatechin content in mature fruit demonstrated clear allelic association between trait and allelotype.

The largest and best‐studied class of polyphenols are the flavonoids; however, previous reports of QTLs associated with flavonoid content in peach were limited (Scorza *et al*., 1991), although candidate genes on Chr. 3 and 5 were predicted (Dantec *et al*., 2010). The hot spot for QTLs associated with the total phenol and flavonoid content in peach fruit (Zeballos *et al*., 2016) at 2.5–5.7 Mb on Chr. 2 is distant from the association signals for catechin and epicatechin content at 13.72–13.77 Mb on Chr. 2 identified in the present study, making our contribution a significant addition to the body of knowledge on the control of polyphenol content in peach fruit.

We found that the allelotype of SNPs associated with the 171 individual phenotypic traits associated with fruit weight or the acid, sugar or polyphenol content changed during evolution from wild related species to improved varieties of peach (Figure S29). Notably, the trend was towards heterozygosity for fruit weight, SSC/TA and acid content, but towards homozygosity for both sugar and polyphenol contents. Changes in the content of polyphenolic compounds may have evolved through unconscious negative selection when our ancestors imposed intense selective pressures for fruit size and high sugar content. We found that alleles associated with increased polyphenol content comprised a very low proportion in landraces and were virtually absent in improved varieties. This result can be compared with results in tomato, in which researchers found that modern commercial varieties contain significantly lower amounts of many important chemical components than older varieties (Tieman *et al*., 2017).

### Breeding prospects

The information presented here on the allelic status at loci associated with fruit weight as well as the acid, sugar and polyphenol contents provides valuable information for peach breeders. The trend towards heterozygosity in fruit weight indicates that there is much room for the improvement of this trait, a proposition supported by the conclusion of Velasco *et al*. (2016) that fruit weight in peach was selected for prior to species divergence as well as the finding that several QTLs in wild species contribute favourably to fruit size in peach (Quilot *et al*., 2004). The heterozygous nature of the association SNPs for acidity will enable breeders to finely manipulate this trait to maintain the optimal sugar/acid balance that is necessary for flavour enhancement in improved varieties. There is still a little room to move towards greater sweetness. Finally, we suggest that higher levels of health‐related polyphenols in improved varieties could be recovered through distant hybridization with wild related species aided by genomic selection. However, it should be noted that we should select for specific potent compounds with a high nutritional value that do not lead to bitterness (Peleg *et al*., 1999) and browning (Gradziel and Wang, 1993) in fruit.

## Conclusions

We have dissected the genetic status of QTLs associated with the control of several important fruit quality traits and investigated their evolutionary status. Our study generated a large data set of QTLs and candidate genes related to the domestication and improvement of peach. The genome resources we have developed in this study allowed the large‐scale characterization of key agronomic traits in peach as well as a genomic repertoire for evolutionary research. We believe that further investigation of this data set will help to determine the underlying mechanisms of peach evolution and provide a foundation for the further genetic improvement of peach.

## Materials and methods

### Selection of plant material

In total, 418 accessions (Table S1) were chosen to represent the entire range of phenotypic diversity and geographic distribution of the peach. Among them, 93 varieties had been used in a previous study (Cao *et al*., 2016). A phylogenetic tree constructed using genomic sequencing data (see Population genetics analysis section below) was employed to exclude varieties with an admixed structure, leaving 323 accessions that were used to identify selection sweeps. For GWAS, wild related species, wild peach and ornamental peach were excluded to achieve fine control of population structure.

The roots, phloem, mature leaves, mature fruit and seeds of three varieties belonging to wild related species (‘A Ba Gung He Tao’, ‘Zhou Xing Shan Tao’ and ‘Hong Gen Gan Su Tao’); three ornamental peaches (‘Hong Ye Chui Zhi’, ‘Shou Bai’ and ‘S1’); three edible landraces (‘Chinese Cling’, ‘Nanshan Tian Tao’ and ‘Huo Lian Jin Dan’); and three improved varieties (‘Yu Lu’, ‘Fantasia’ and ‘96‐2‐51’) were selected for total RNA sequencing to study the tissue‐specific expression of genes under selection.

Because of their contrasting sugar and polyphenol content, ‘Tianjin Shui Mi’ and ‘Hakuho’ were utilized to screen the expression of candidate genes for fruit weight and sorbitol, catechin and epicatechin content underlying the QTLs on Chr. 6, Chr. 4 and Chr. 2 that were identified in the study. Fruit was collected at six developmental stages (24 April, 8 May, 24 May, 8 June, 24 June and 8 July 2014) for the RNA‐Seq study, and the sorbitol, sucrose, glucose, fructose, catechin and epicatechin contents were measured on 8 May, 24 May, 8 June, 24 June and 8 July 2014. The IAA concentration was measured in the ‘Tianjin Shui Mi’ peach 20, 40, 60, 80 and 100 days after full bloom.

The 75 varieties used to clone the promoter and gene sequence of the fruit weight candidate gene *Prupe.6G046800* are listed in Table S14.

For bulked segregant RNA‐Seq analysis of the candidate genes underlying the QTL for sorbitol content on Chr. 4, 30 peach varieties from two groups with a different sorbitol content (low and high) in their mature fruit were chosen (Table S15).

The above accessions were all maintained at the National Fruit Tree Germplasm Repository, Zhengzhou Fruit Research Institute, Chinese Academy of Agricultural Sciences, China, in sandy loam soils. The trees were 12 years old and had been propagated by bud grafting onto ‘Xinjiang Mao tao’ rootstocks (*P*. *persica* with small fruit, from Sinkiang Province). The trees were trained on a V‐shaped system with spacing of 2 m within and 5 m between rows. Pruning treatments were applied annually at bud break. The detailed parameters are as follows: no pruning in spur branches shorter than 10 cm, with three to five flower buds retained in those branches with 10–25 cm between them and 6–8 flower buds retained in branches longer than 25 cm. Sometimes light pruning was performed in the summer to open the canopy and improve tree vigour and fruit quality. The fruits were thinned after the first physiological fruit drop to a load of ~50 fruit per tree according to vigour in order to allow the full expression of fruit size without limitation due to competition. Field management protocols during the growing period, including irrigation, fertilizing and pruning, were carried out under standard conditions for all accessions. Fruits from each accession were harvested when their growth had stopped, they began softening, and they exhibited a yellow or orange ground colour.

### Phenotyping

The fruit and stone shapes of the peaches were measured using vernier callipers in 2014. The fruit weight and SSC were recorded for 323 varieties (Table S1) using an electronic balance (Quintix313‐1CN; Sartorius, Gottingen, Germany) and a refractometer (PAL‐1; Atago, Tokyo, Japan), respectively, in five fruit from each maturity date in 2014, 2015 and 2016. The TA was determined to calculate the SSC/TA ratio through manual titration with a 0.1 m NaOH solution and using a phenolphthalein indicator until an end point with a constant pH (8.0 ± 0.1). Flesh adhesion and flesh texture were analysed after the maturity date only in 2014 (Table S1) based on evaluation criteria used for previously published plant genetic resources (Wang and Zhu, 2005).

Four sugar (fructose, glucose, sorbitol and sucrose) and acid (citric acid, malic acid, quininic acid and succinic acid) components were quantitated over three years (2013, 2014 and 2015) in mature fruit from the 313 varieties of peach (Table S10 and Figures S6, S26).

At each sampling date, duplicate samples of powdered flesh (2.0 g) from five fruit frozen with liquid nitrogen were mixed with 8 mL of a water : ethanol solution (20 : 80, vol/vol) for 30 min in a 10‐mL centrifuge tube. To increase cell disruption and solute extraction, the tubes were then placed in a water bath for 20 min at 35 °C. The solutions were centrifuged at 4500 ***g*** at 4 °C for 15 min, the supernatants were transferred to a vial, and the residue was further extracted with 8 mL of the same solution. Both supernatants were mixed together and brought up to a final volume of 25 mL. Then, the solution was purified at 40 °C using a rotary evaporator until all ethanol was removed. One milliliter of ultrapure water was added to the residue, mixed with a vortex mixer, transferred to an Eppendorf tube and filtered through a 0.22‐μm nylon filter before analysis.

The individual sugar (fructose, glucose, sorbitol and sucrose) contents were determined in three technical replicates using a high‐performance liquid chromatography (HPLC) system (Model 2695; Waters, Milford, MA) equipped with a refractive index (RI) detector (Model 2414; Waters) and a Venusil XBP‐NH2 column (4.6 mm × 250 mm, Bonna‐Agela Technologies, Tianjin, China). The mobile phase was ultrapure water : acetonitrile (18 : 82, vol/vol) at a flow rate of 1 mL/min. Sugars were identified by comparison of the relative retention times of sample peaks with those of external standards. Pure analytical standards for d‐(+)‐glucose, d‐(−)‐fructose, sucrose and d‐sorbitol were purchased from Sigma‐Aldrich (St. Louis, MO). The results were expressed as mg/g (fresh sample), calculated by internal normalization of the chromatographic peak area and application of individual calibration curves. The citric acid, malic acid, quininic acid and succinic acid contents were determined in three technical replicates by ion chromatography (Model 861861; Metrohm, Herisau, Switzerland) with an ICSep Coregel column using the above extraction solution. The optimized column temperature was 65 °C, and the mobile phase was a 2.5 mol/L H_2_SO_4_ solution with a flow rate of 0.7 mL/min.

To determine the catechin, epicatechin, chlorogenic, neochlorogenic acid and procyanidin B1 content in fruit from the 313 varieties of peach sampled in 2014 and 2015, 5 g samples of fruit flesh powder were placed in a 50‐mL tube with 8 mL of 2% formic acid/methanol, ultrasonicated for 20 min (25 °C, 40 Hz, 100 W) and then centrifuged for 8 min (25 °C; 12 000 ***g***); the above steps were repeated three times, and the supernatants were combined. Twenty millilitres of this solution was evaporated and redissolved to a volume of 5 mL using 2% formic acid/methanol and stored at −80 °C until assayed. A 3 mL aliquot of the polyphenol extract was removed for evaporation of the methanol organic phase using a rotary evaporator at 40 °C. The remaining aqueous phase was transferred to an activated solid‐phase extraction cartridge. The filtrate was eluted with 3 mL of methanol (containing 0.05% HCl) twice and filtered through a 0.22‐μm nylon filter before analysis. The polyphenol content was measured in three technical replicates using ultra‐high‐performance liquid chromatography (UPLC)–mass spectrometry (MS) (Waters) with Waters Acquity UPLC^®^ HSS T3 columns (2.1 × 150 mm). The column temperature was 40 °C, the injection volume was 2.0 μL, and the elution gradient was 0.5% formic acid solution and acetonitrile at a flow rate of 0.3 mL/min. We set the conditions for electrospray ionization–MS as follows: ion source temperature = 150 °C, desolvation temperature = 400 °C and desolvation flux = 800 L/h.

To analyse the sorbitol, sucrose, glucose, fructose, catechin, epicatechin and IAA content in developing fruit from the ‘Hakuho’ and ‘Tianjin Shui Mi’ varieties of peach (S1–S6, corresponding to 30, 45, 60, 75, 90, 105 days after full bloom), 100 mg dried powdered flesh was extracted overnight at 4 °C in 1.0 mL of 70% aqueous methanol containing 0.1 mg/L lidocaine (internal standard), and the solution was centrifuged at 10 000 ***g*** for 10 min. An 0.4 ml aliquot of the supernatant was filtered before analysis using high‐throughput liquid chromatography (LC)‐MS/MS (Chen *et al*., 2013). The qualification of metabolites was carried out using a multiple reaction monitoring procedure.

### DNA resequencing

Total genomic DNA was extracted from the young leaves of 418 germplasm accessions using the CTAB (hexadecyl trimethyl ammonium bromide) method (Murray and Thompson, 1980). The libraries were constructed with an insert size of 500 bp and sequenced on an Illumina HiSeq X Ten platform (Illumina, San Diego, CA) in paired‐end (PE) mode with a 150 bp read length by Annoroad Gene Technology (Beijing, China).

### RNA sequencing and analysis of expression levels

Total RNA was extracted from the plant tissues specified in the individual experiments using an RNA Extraction Kit (Aidlab, Beijing, China), following the manufacturer's protocol. Magnetic beads with oligo(dT) were used to enrich for mRNA, and double‐stranded cDNA was synthesized following the addition of buffer solution, dNTPs, RNase H and reverse transcriptase I. We then added a single ‘A’ base to the cDNA fragments and ligated the adapter. The products were then purified and enriched using PCR amplification. The resulting cDNA libraries were sequenced on a HiSeq 2500 system (Illumina) in PE mode with a 100 bp read length. More than 6 GB of data was generated for each sample.

After filtering the adapter from the sequencing reads, the data were aligned against the peach reference genome v2.0 (Verde *et al*., 2017) using TopHat v2.1.0 (Kim *et al*., 2013). Gene expression information was calculated as the FPKM using Cufflinks v2.1.1 (Trapnell *et al*., 2010) and RNA‐Seq by Expectation Maximization (RSEM) v1.2.11 (Li and Dewey, 2011).

### Detection of variants

First, the raw data were processed with Perl scripts to ensure the quality of the data used in further analysis. The filtering criteria used were as follows: (i) adaptor‐polluted reads containing more than 5 adapter‐polluted bases were removed, (ii) reads with low‐quality bases (Phred quality value < 19) accounting for more than 50% of the total bases were removed, and (iii) reads with N bases accounting for more than 5% of the total bases were removed. For paired‐end sequencing data, both reads were filtered out if eight read of the paired‐end reads was adaptor‐polluted. Second, the paired‐end reads of all resequenced accessions described above were aligned against the peach reference genome v2.0 (Verde *et al*., 2017) using the program Burrows–Wheeler Aligner (BWA) v0.7.12 (Li and Durbin, 2009). SAMtools v1.2 (Li *et al*., 2009) was used to sort reads, and duplicate reads from PCR were removed using Picard Tools v1.13 (http://broadinstitute.github.io/picard/) MarkDuplicates. Reads mapping to two or more places were filtered out. Third, the SNPs were called using the Genome Analysis Toolkit v3.4 (GATK; McKenna *et al*., 2010) HaplotypeCaller protocol via local reassembly of haplotypes for the population. The SNPs were filtered out before analysis with the GATK VariantFiltration protocol. The filtering settings were as follows: QD < 10, FS > 10.0, DP < 4 and QUAL < 30. Annotation was then performed using ANNOVAR v2014‐07‐14 (Wang *et al*., 2010) for all the qualified variants based on the GFF file. To validate SNPs, we evaluated 22 SNPs and 138 genotypes using Sequenom MassARRAY technology (http://www.sequenom.com, Data S1 and Table S3).

A total of 4 236 364 single nucleotide polymorphisms (SNPs) initially detected among the 418 accessions were filtered out by discarding SNP sites with a minor allele frequency (MAF) smaller than 0.02 and genotypes called below 20% across all individuals. A total of 2 830 238 high‐quality common SNPs were retained for downstream analysis. Among them, a total of 313 accessions were used for GWAS with 988 306 polymorphic SNPs.

### Population genetics analysis

Genetic differentiation between different populations was assessed using *F*_ST_ (Weir and Cockerham, 1984) and calculated using a previously described method (Cao *et al*., 2014).

Phylogenetic trees were constructed using Phylogeny Inference Package (PHYLIP) v3.2 software (Felsenstein, 1989) with 1000 bootstraps in a subset of 46 562 SNPs from the 418 peach accessions, selected randomly from the SNP data set. Trees were visualized from the distance matrix with Molecular Evolutionary Genetics Analysis (MEGA) v5.0 software (Tamura *et al*., 2011). The population structure was investigated in the same data set, using STRUCTURE v2.3.1 (Pritchard *et al*., 2000) based on allele frequencies. To determine the most likely group number, the initial burn‐in period was set to 50 000, with 100 000 Markov chain Monte Carlo iterations with *K* values from 2 to 8. Then, Δ(*K*) was calculated according to the methods of Evanno *et al*. (2005).

In addition, we performed PCA using Genome‐wide Complex Trait Analysis v1.25.3 (Yang *et al*., 2011) based on 2 785 339 SNPs with a MAF > 0.05 and a missing rate < 10%. Two‐dimensional coordinates were plotted for the sets of 418 and 323 peach accessions using Microsoft^®^ Excel v2010.

### GWAS

Association analysis of fruit weight and the sugar, acid and polyphenol contents in fruit was conducted for 988 306 filtered SNPs from 313 accessions using the linear mixed model in FaST‐LMM v2.07 software (Lippert *et al*., 2011). The Q matrix was evaluated using STRUCTURE v2.3.1 software (Pritchard *et al*., 2000). The pair‐wise kinship (*K*) coefficients between the accessions were estimated in FaST‐LMM v2.07 software (Lippert *et al*., 2011) for all SNPs. A stringent Bonferroni correction was used to screen obvious association signals based on *P* value (significant genome‐wide threshold: ‐log_10_ *P* ≥ 7.29) calculated by dividing 0.05 by 988 306 SNPs. Manhattan and quantile–quantile plots were generated in R v3.5.1 using the package qqman v0.1.2 (Turner, 2014).

### Identification of potential candidate genes

As the linkage disequilibrium decay in peach was approximately 20~50 kbp at the genome‐wide level (Cao *et al*., 2014), candidate genes were identified within a 50‐kb window spanning loci identified by GWAS, using the peach reference genome v2.0 (Verde *et al*., 2017). These potential candidates were evaluated using expression analysis (see above).

### Candidate gene resequencing

We designed primers to clone the promoter and coding regions of the candidate gene *Prupe.6G046800* for fruit weight using the peach reference genome v2.0 (Verde *et al*., 2017) and Primer‐BLAST software (http://www.ncbi.nlm.nih.gov/tools/primer-blast/). The PCR products from 75 varieties were sequenced using traditional Sanger technology and assembled manually. A total of 64 SNPs were identified and examined for obvious associations with fruit weight in the years 2015 and 2016.

### Bulked segregant RNA‐Seq analysis of candidate genes for sorbitol content in peach fruit

To identify candidate genes located under the QTL for sorbitol content on Chr. 4 identified by GWAS, we calculated the relative expression of 180 selected genes using two bulked RNA extracted from the fruit of 15 low sorbitol‐containing accessions and 15 high sorbitol‐containing accessions using RNA‐Seq (a total of 30 varieties were sequenced separately with one biological replicate). The genes with differential expression between the two bulks were selected for further analysis.

### Detection of genomic regions subjected to selection and allele frequency divergence

To identify genomic regions affected by domestication, the level of genetic diversity (π) between wild related species and varieties of *P. persica* (wild peach, ornamental peach, edible landraces and improved varieties) was calculated using a 50‐kbp window with a step size of 10 kb in the vcftools v0.1.12b package (Danecek *et al*., 2011). The improvement‐related regions were then screened based on the ratio of the π values between edible landraces and the improved varieties. When calling selective sweeps, if the π of wild peach together with that of edible landraces and improved varieties for a window was lower than 0.002 in the domestication analysis and the π of improved varieties was lower than 0.001 in the improvement analysis, then the window was excluded. By scanning the domestication‐ and improvement‐related sweeps, we selected windows with the top 5% of ratios (9.06 and 3.84 for domestication and improvement, respectively) as candidate regions for further analysis. Continuous windows longer than 100 kbp were merged into a single region. In addition, cross‐population extended haplotype homozygosity analysis (XP‐EHH) was applied to detect strong recent selection sweeps (Olsen and Wendel, 2013) using selscan v1.2.0a software (Szpiech and Hernandez, 2014). We used wild related species as references for domestication simulations in wild and ornamental peach, landraces and improved varieties; the landraces were used as the reference for improvement simulations in improved varieties. The detailed procedure used is described below as follows: SNPs with a MAF < 0.05 were filtered out. The average genetic distance was obtained from the reference linkage map of peach (Howad *et al*., 2005). SHAPEIT v2.644 (Delaneau *et al*., 2012) phased haplotypes with 50‐kbp windows with 10 kbp steps. We computed XP‐EHH scores using selscan v1.2.0a based on the phased haplotypes. The XP‐EHH score of each variant was standardized by the mean XP‐EHH and the standard deviation over the entire genome. Dotted lines in XP‐EHH figures delineate the genome‐wide upper 5% quantile of SNPs. To cast light on the underlying cause behind the formation of selection sweeps, Kyoto Encyclopedia of Genes and Genomes (KEGG, Kanehisa *et al*., 2002) enrichment analysis of these candidate genes was performed using the NCBI blastall program (ftp://ftp.ncbi.nih.gov/blast/executables/).

### Tissue‐specific expression pattern of genes under selection

To identify the key selection genes located in the respective sweep regions, five tissue types in the four groups of peaches (each comprising three biological replicates) were used for RNA‐Seq analysis. Reads containing adapters or low‐quality sequences were removed (Bolger *et al*., 2014) before aligning clean reads to the peach reference genome v2.0 (Verde *et al*., 2017) using TopHat v2.1.0 (Kim *et al*., 2013). Gene expression levels were calculated as FPKM values and depicted in heat maps for the genes under selection using the R package pheatmap (https://cran.r-project.org/web/packages/pheatmap/index.html). Candidate genes with a low expression rate (average FPKM in three replicates < 1) were filtered out and the logFC (log fold change) of differential expression (DE) ≥ 2 when ornamental and wild peach were compared to wild related species (domestication) and when improved varieties were compared to landraces (improvement) were calculated.

### Transgenic analysis

The full‐length open reading frames of the *Prupe.6G046800* gene were amplified through PCR using cDNAs synthesized from RNA that was isolated from fruit of the ‘Tianjin Shui Mi’ peach. The amplified products were further cloned into the pHELLSGATE8 vector driven by the cauliflower mosaic virus (CaMV) 35 S promoter. The resulting constructs and empty vectors that served as controls were further transformed into *Solanum lycopersicum* cv. Ailsa Craig tomato by *Agrobacterium tumefaciens* C58. The T0 plants were generated and grown to maturity in pots under glasshouse conditions to observe plant height and measure gene expression.

### Data access

All raw sequence reads of the 418 peach accessions have been deposited in the Sequence Read Archive (SRA) of National Center for Biotechnology Information with accession numbers SRP108113, SRP168153 and SRP173101. Detailed information for each accession is listed in Table S1.

## Conflict of interest

The authors declare there is no conflict of interest.

## Author contributions

L.W. and K.C. conceived the project and its components. G.Z., W.F., C.C. and D.M. collected samples and performed phenotyping. Y.L. and X.W. conducted gene expression analysis. K.C. analysed all the data. K.C., C.H.D. and S.E.G. wrote the paper. All authors read and approved the final manuscript.

## Supporting information


**Figure S1** The distribution of MAF for SNP derived from resequencing of 418 accessions.
**Figure S2** Enlarged view of wild related group in the phylogenetic tree of 418 accessions (Figure 1b).
**Figure S3** Enlarged view of improved variety subgroup in the population structure of 418 accessions (Figure 1c) when *K* = 4 (a) and its population (b).
**Figure S4** The genomic polymorphism calculated using π value of improved varieties coming from China (a), Japan and South Korea (b), Europe and America (c) are plotted against position on each of the chromosomes.
**Figure S5** The distribution of selection sweeps associated with domestication and improvement among the eight chromosomes (Chr.) of peach.
**Figure S6** Frequency distribution of the variation in several traits across the 313 peach fruit samples used in the genome‐wide association study.
**Figure S7** Genome‐wide association study for fruit vertical diameter for 313 landraces and improved varieties using FaSTLMM software with the mixed linear model (MLM) comprised the kinship value (*K*) that (a) did not control for population structure (Q) or controlled it (b).
**Figure S8** Genome‐wide association study for (a) fruit cheek diameter and (b) fruit suture diameter for 313 landraces and improved varieties using FaSTLMM software.
**Figure S9** Genome‐wide association study for (a) stone length, (b) stone width, and (c) stone thickness for 313 landraces and improved varieties using FaSTLMM software.
**Figure S10** Genome‐wide association study for (a) fresh stone weight and (b) flesh/pit ratio for 313 landraces and improved varieties using FaSTLMM software.
**Figure S11** Genome‐wide association study for (a) flesh adhesion and (b) flesh texture for 313 landraces and improved varieties using FaSTLMM software.
**Figure S12** Genome‐wide association study for fruit weight traits in (a) 2014, (b) 2015, and (c) 2016 for 313 landraces and improved varieties using FaSTLMM software.
**Figure S13** Genome‐wide association study for soluble solids content in fruit in 2014 (a), 2015 (b), and 2016 (c) for 313 landraces and improved varieties using FaSTLMM software.
**Figure S14** Genome‐wide association study for the soluble solids content—titratable acidity ratio in fruit in 2014 (a), 2015 (b), and 2016 (c) for 313 landraces and improved varieties using FaSTLMM software.
**Figure S15** Genome‐wide association study for citric acid content in fruit in (a) 2013, (b) 2014, and (c) 2015 for 313 landraces and improved varieties using FaSTLMM software.
**Figure S16** Genome‐wide association study for malic acid content in fruit in (a) 2013, (b) 2014, and (c) 2015 for 313 landraces and improved varieties using FaSTLMM software.
**Figure S17** Genome‐wide association study for quininic acid content in fruit in (a) 2013, (b) 2014, and (c) 2015 for 313 landraces and improved varieties using FaSTLMM software.
**Figure S18** Genome‐wide association study for succinic acid content in fruit in (a) 2013, (b) 2014, and (c) 2015 for 313 landraces and improved varieties using FaSTLMM software.
**Figure S19** Genome‐wide association study for fructose content in fruit in (a) 2013, (b) 2014, and (c) 2015 for 313 landraces and improved varieties using FaSTLMM software.
**Figure S20** Genome‐wide association study for glucose content in fruit in (a) 2013, (b) 2014, and (c) 2015 for 313 landraces and improved varieties using FaSTLMM software.
**Figure S21** Genome‐wide association study for sorbitol content in fruit in (a) 2013, (b) 2014, and (c) 2015 for 313 landraces and improved varieties using FaSTLMM software.
**Figure S22** Genome‐wide association study for sucrose content in fruit in (a) 2013, (b) 2014, and (c) 2015 for 313 landraces and improved varieties using FaSTLMM software.
**Figure S23** Genome‐wide association study for catechin content in fruit in (a) 2014 and (b) 2015, epicatechin content in fruit in (c) 2014 and (d) 2015, chlorogenic content in fruit in (e) 2014 and (f) 2015, neochlorogenic content in fruit in (g) 2014 and (h) 2015, procyanidin B1 content in fruit in (i) 2014 and (j) 2015 for 313 landraces and improved varieties, which were performed using FaSTLMM software.
**Figure S24** Correlation analysis among the traits of peach evaluated in the study.
**Figure S25** The phenotypic distribution of flesh adhesion (a) and texture (c) in different populations and subpopulations (b and d) of improved varieties.
**Figure S26** The evolution of peach with respect to appearance (a), fruit weight and soluble solids content (b). Data were derived from analysis of 323 accessions.
**Figure S27** The content of 4 acid, 4 sugar, and 5 polyphenol related fruit compounds in landraces and improved varieties over different years of sampling.
**Figure S28** Results of the Cross Population Extended Haplotype Homozygosity (XP‐EHH) analysis to detect selection sweeps under domestication (a) and improvement (b). In the panel, the dotted line represents the upper 5% quantile of XP‐EHH values. The QTLs which overlapped with the selection sweeps are indicated with arrows.
**Figure S29** The change in allele frequency for the SNP most highly associated with a range of trait loci in different peach populations. The number in the left column indicates the locus accession in Table S11.

**Table S1** Peach samples used in this study and their sequencing results
**Table S2** The accession‐specific SNP in different population
**Table S3** Distribution of SNPs in 8 chromosomes of peach
**Table S4** Validation of SNPs using the Sequenom MassARRAY platform
**Table S5** Levels of genetic differentiation (*F*_ST_) in different populations
**Table S6** Identification of selection sweeps for domestication and improvement calculated by two methods
**Table S7** Comparison of the reported domestication QTLs with domestication sweeps identified in this study
**Table S8** The physical location of highly differentiated sweep regions associated with domestication and improvement in peach
**Table S9** The physical location of large‐effect sweep regions associated with domestication and improvement in peach
**Table S10** The summarized phenotypic data set for quantitative traits in different years
**Table S11** Genome‐wide association signals of agronomic traits for 313 peach accessions in different years
**Table S12** The candidate genes located in the QTL for fruit weight on chromosome 6
**Table S13** The expression (FPKM value) of candidate genes located under the QTL for fruit weight during the fruit development period of ‘Tianjin Shui Mi’ peach
**Table S14** The allelotypes of *Prupe.6G046800* associated with fruit weight in 75 accessions
**Table S15** The sorbitol content of varieties used for bulked segregant RNA‐Seq analysis
**Table S16** The χ^2^ test for expression data obtained from high and low fruit sorbitol bulks respectively, for 85 Chromosome 6 candidate genes putatively associated with sorbitol content

**Appendix S1** Candidate genes identified in the sweep regions with the most obvious and large‐effect selection signals.

**Data S1** SNP validation by Sequenom MassArray technology.
