# Supplementary material for: Comparative population genomics identified genomic regions and candidate genes associated with fruit domestication traits in peach
Source: Plant Biotechnol J. 2019 Apr 16;17(10):1954–70. doi: 10.1111/pbi.13112 (PMC6737019; doi:10.1111/pbi.13112)
Supplement: Supplementary file 1 — Figure S1 The distribution of MAF for SNP derived from resequencing of 418 accessions. Figure S2 Enlarged view of wild related group in the phylogenetic tree of 418 accessions (Figure 1b). Figure S3 Enlarged view of improved variety subgroup in the population structure of 418 accessions (Figure 1c) when K = 4 (a) and its population (b). Figure S4 The genomic polymorphism calculated using π value of improved varieties coming from China (a), Japan and South Korea (b), Europe and America (c) are plotted against position on each of the chromosomes. Figure S5 The distribution of selection sweeps associated with domestication and improvement among the eight chromosomes (Chr.) of peach. Figure S6 Frequency distribution of the variation in several traits across the 313 peach fruit samples used in the genome‐wide association study. Figure S7 Genome‐wide association study for fruit vertical diameter for 313 landraces and improved varieties using FaSTLMM software with the mixed linear model (MLM) comprised the kinship value (K) that (a) did not control for population structure (Q) or controlled it (b). Figure S8 Genome‐wide association study for (a) fruit cheek diameter and (b) fruit suture diameter for 313 landraces and improved varieties using FaSTLMM software. Figure S9 Genome‐wide association study for (a) stone length, (b) stone width, and (c) stone thickness for 313 landraces and improved varieties using FaSTLMM software. Figure S10 Genome‐wide association study for (a) fresh stone weight and (b) flesh/pit ratio for 313 landraces and improved varieties using FaSTLMM software. Figure S11 Genome‐wide association study for (a) flesh adhesion and (b) flesh texture for 313 landraces and improved varieties using FaSTLMM software. Figure S12 Genome‐wide association study for fruit weight traits in (a) 2014, (b) 2015, and (c) 2016 for 313 landraces and improved varieties using FaSTLMM software. Figure S13 Genome‐wide association study for soluble solids content in fruit in 2014 [file PBI-17-1954-s003.docx]

Figure S1. The distribution of MAF for SNP derived from resequencing of 418 accessions.


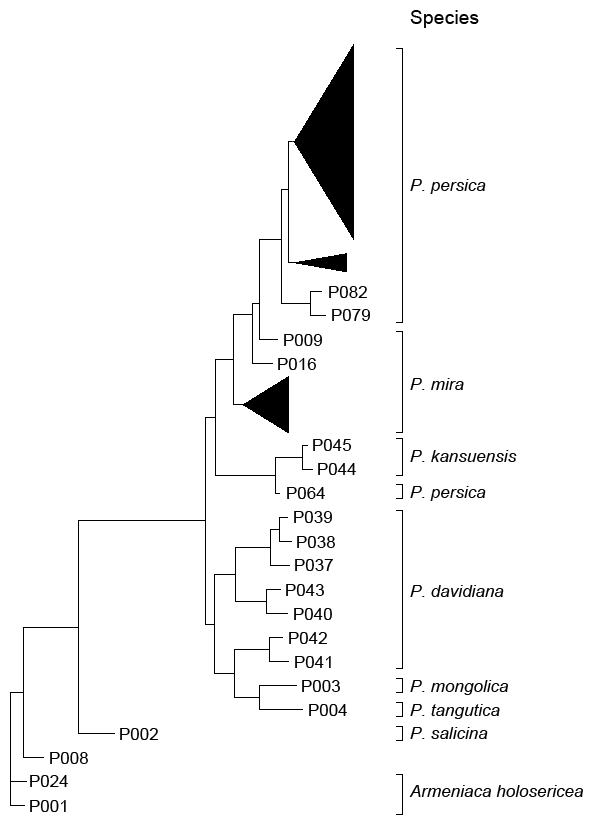


Figure S2. Enlarged view of wild related group in the phylogenetic tree of 418 accessions (Figure 1b).


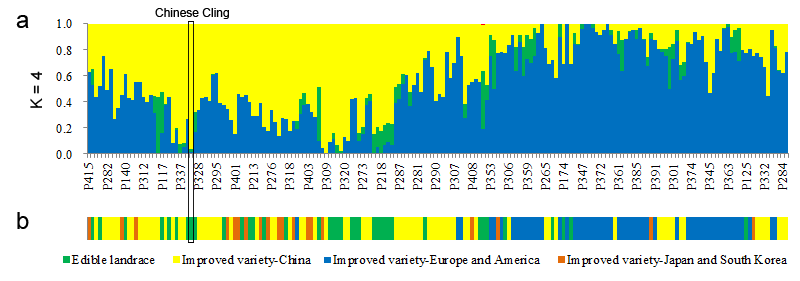


Figure S3. Enlarged view of improved variety subgroup in the population structure of 418 accessions (Figure 1c) when K =4 (a) and its population (b).


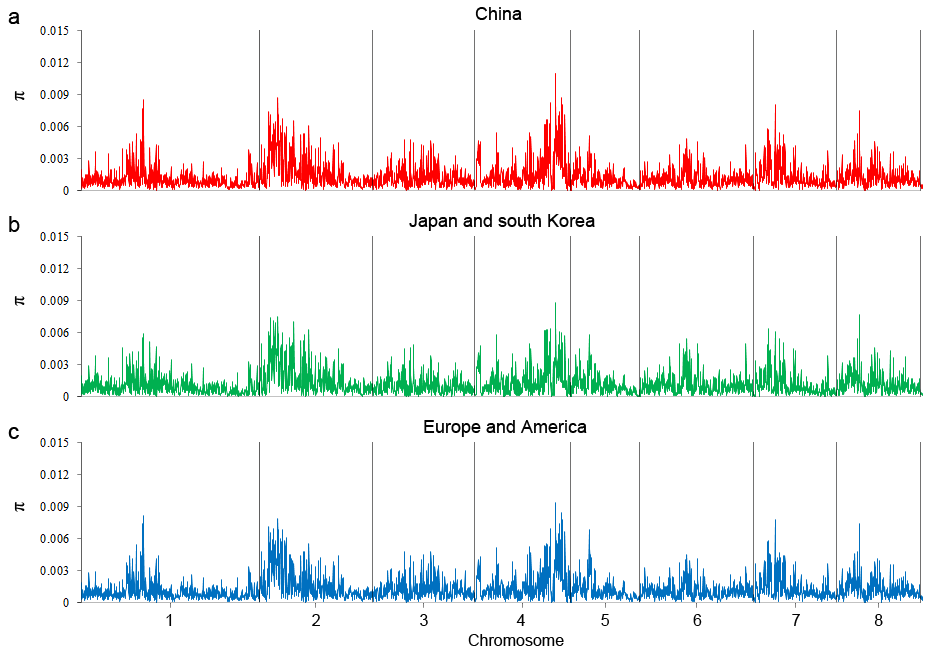


Figure S4. The genomic polymorphism calculated using π value of improved varieties coming from China (a), Japan and South Korea (b), Europe and America (c) are plotted against position on each of the chromosomes.

Figure S5. The distribution of selection sweeps associated with domestication and improvement among the eight chromosomes (Chr.) of peach.

|  |  |  |
| --- | --- | --- |
|  |  |  |
|  |  |  |
|  |  |  |
|  |  |  |
|  |  |  |
|  |  |  |
|  |  |  |
|  |  |  |
|  |  |  |
|  |  |  |
|  |  |  |
|  |  |  |
|  |  |  |
|  |  |  |
|  |  |  |
|  |  |  |
|  |  |  |

Figure S6. Frequency distribution of the variation in several traits across the 313 peach fruit samples used in the genome-wide association study.


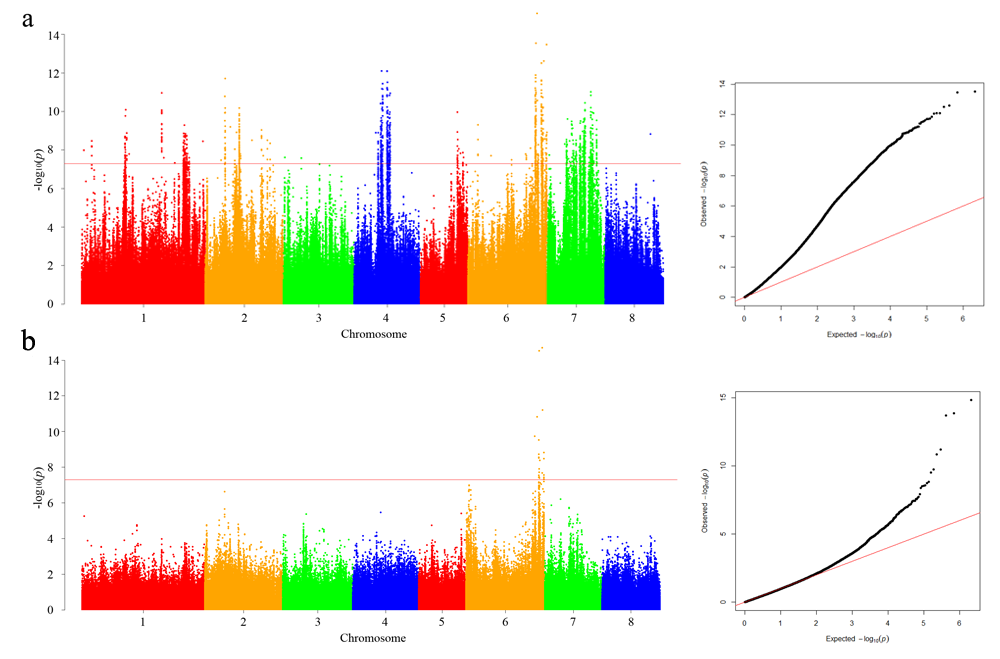


Figure S7. Genome-wide association study for fruit vertical diameter for 313 landraces and improved varieties using FaSTLMM software with the mixed linear model (MLM) comprised the kinship value (K) that (a) did not control for population structure (Q) and considered it (b). Based on the Q‐Q plots, P values of most loci calculated using the MLM (K) method (a) were lower [-log10 (P values) higher] than genome-wide threshold, indicating false positives. Then, we decided to use the results obtained with the MLM (Q + K) model (b) in the following analysis.


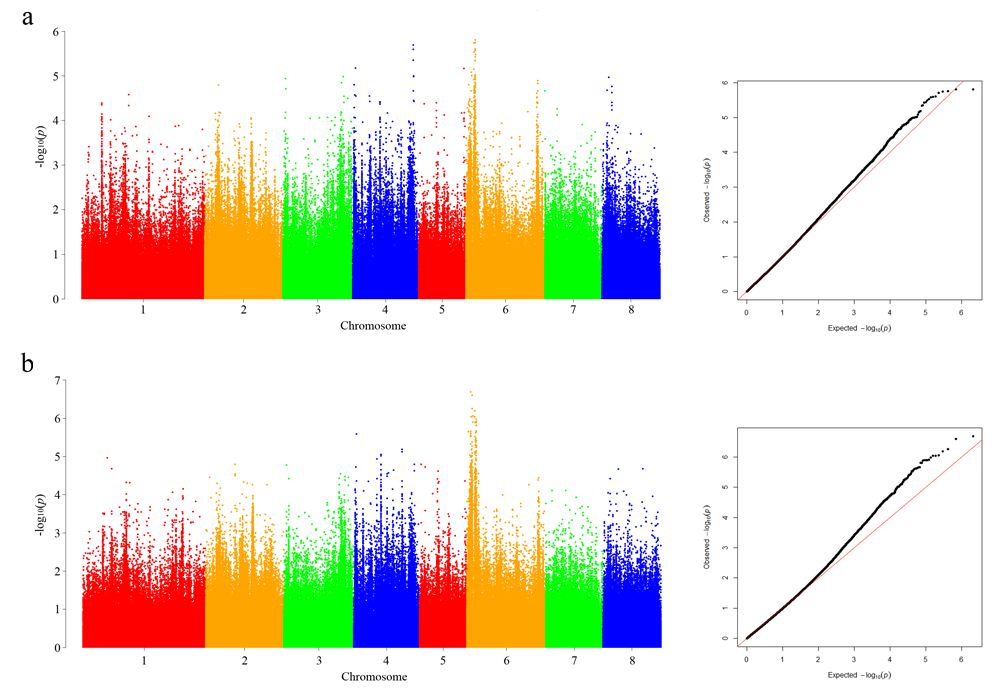


Figure S8. Genome-wide association study for (a) fruit cheek diameter and (b) fruit suture diameter for 313 landraces and improved varieties using FaSTLMM software.


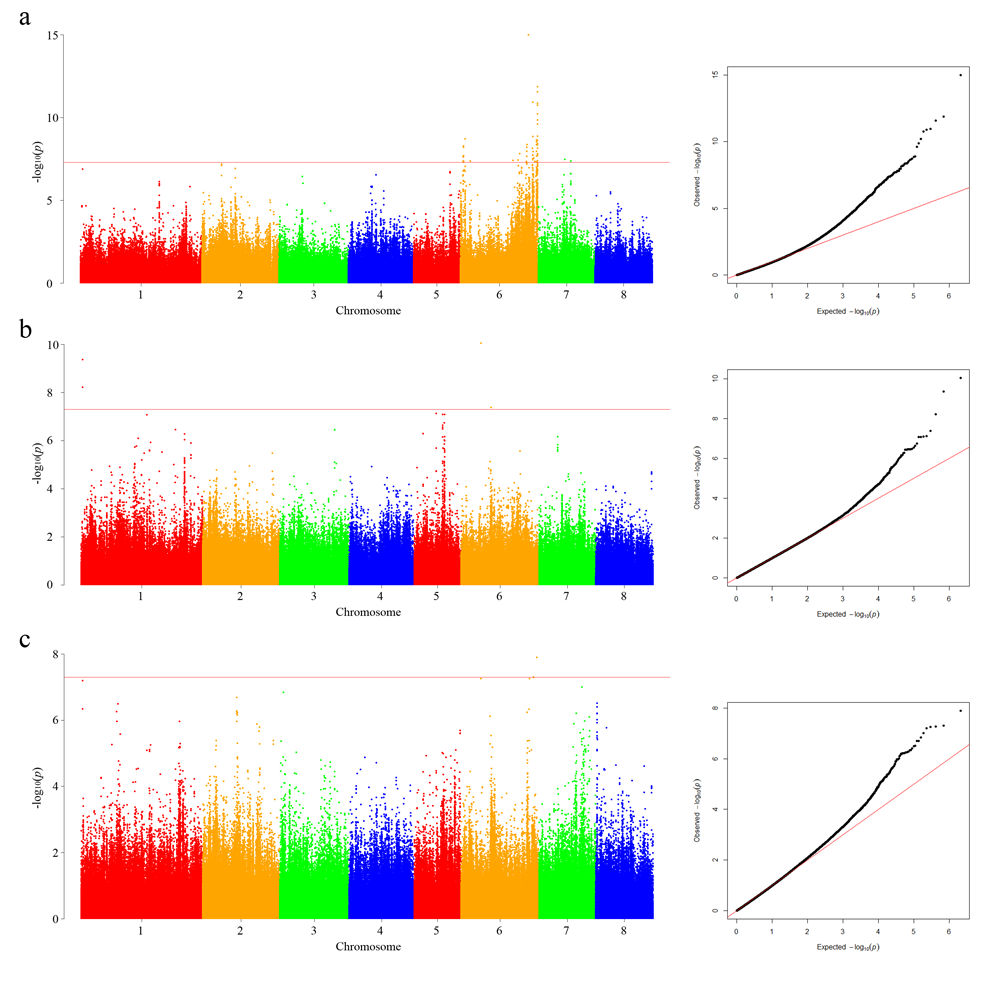


Figure S9. Genome-wide association study for (a) stone length, (b) stone width, and (c) stone thickness for 313 landraces and improved varieties using FaSTLMM software.


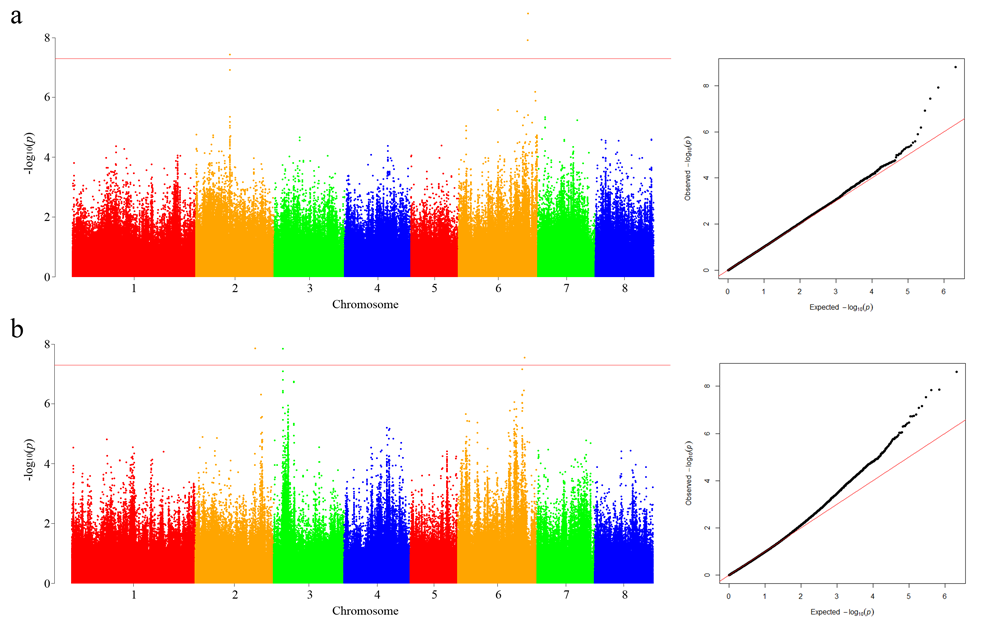


Figure S10. Genome-wide association study for (a) fresh stone weight and (b) flesh/pit ratio for 313 landraces and improved varieties using FaSTLMM software.


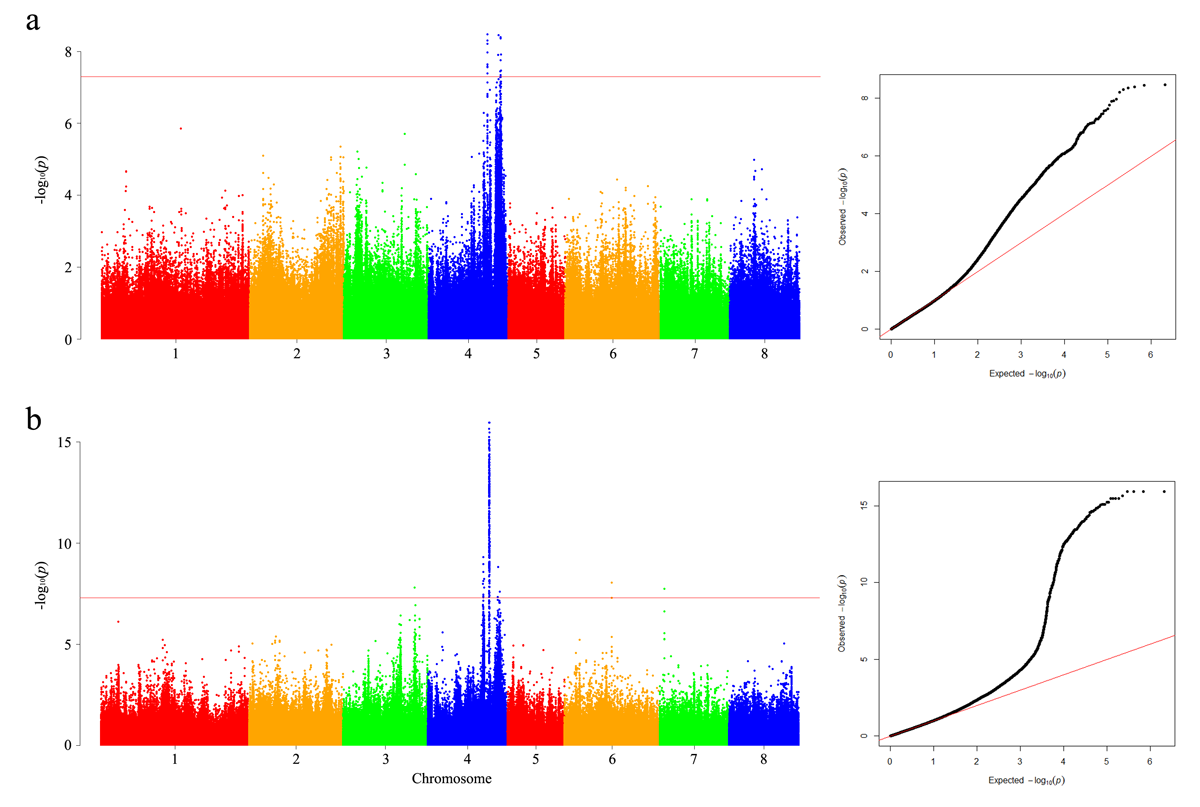


Figure S11. Genome-wide association study for (a) flesh adhesion and (b) flesh texture for 313 landraces and improved varieties using FaSTLMM software.


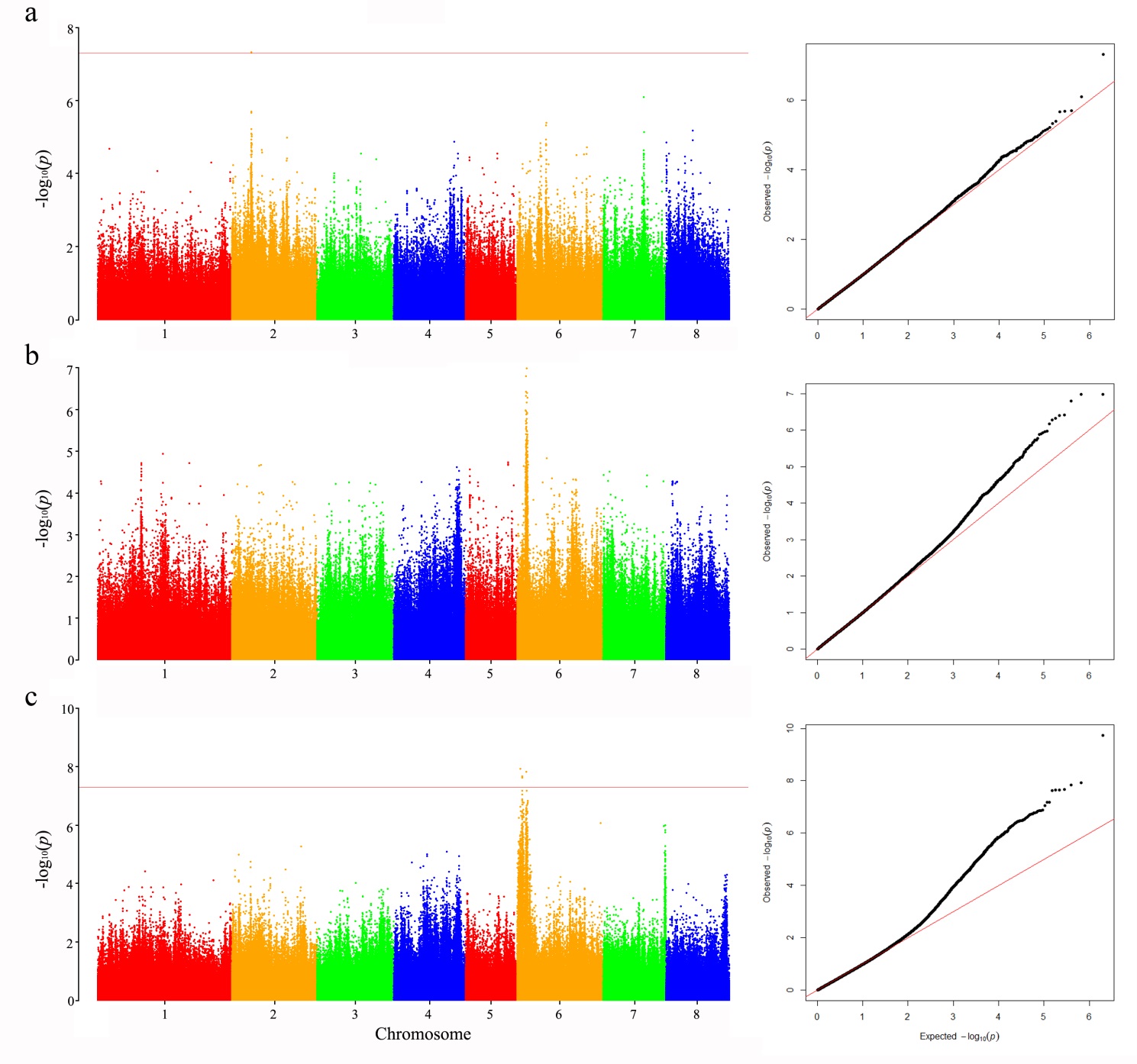


Figure S12. Genome-wide association study for fruit weight in (a) 2014, (b) 2015, and (c) 2016 for 313 landraces and improved varieties using FaSTLMM software.


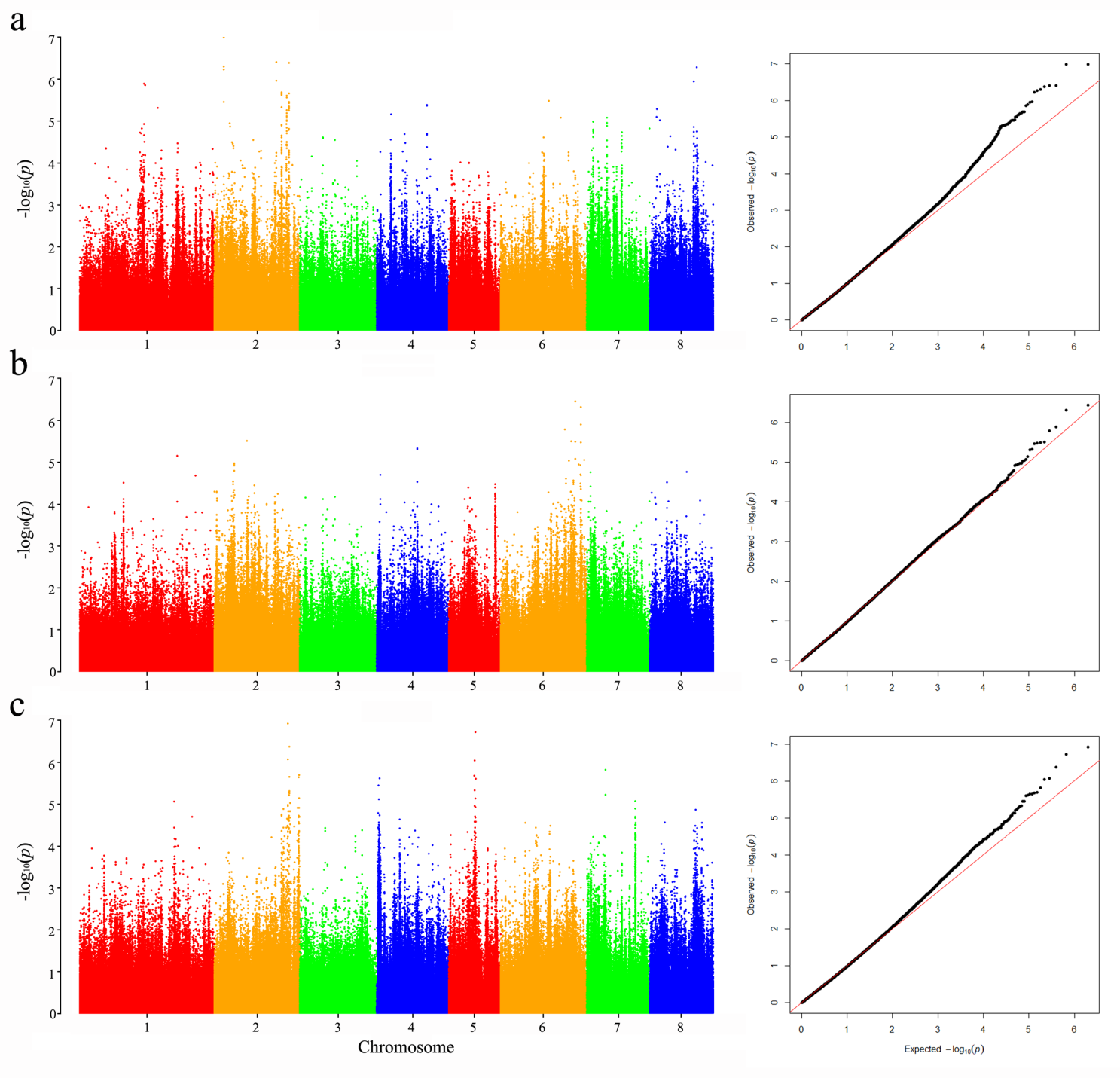


Figure S13. Genome-wide association study for soluble solids content in fruit in 2014 (a), 2015 (b), and 2016 (c) for 313 landraces and improved varieties using FaSTLMM software.


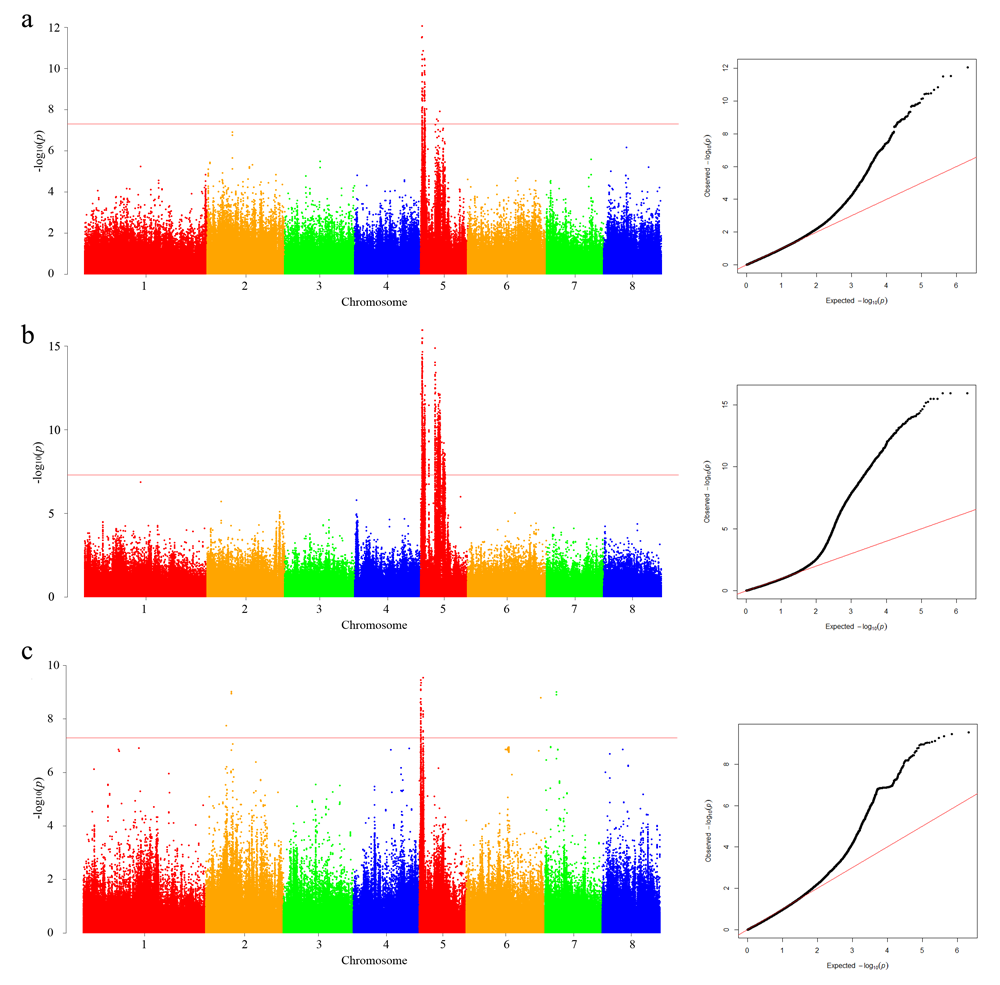


Figure S14. Genome-wide association study for the soluble solids content - titratable acidity ratio in fruit in 2014 (a), 2015 (b), and 2016 (c) for 313 landraces and improved varieties using FaSTLMM software.


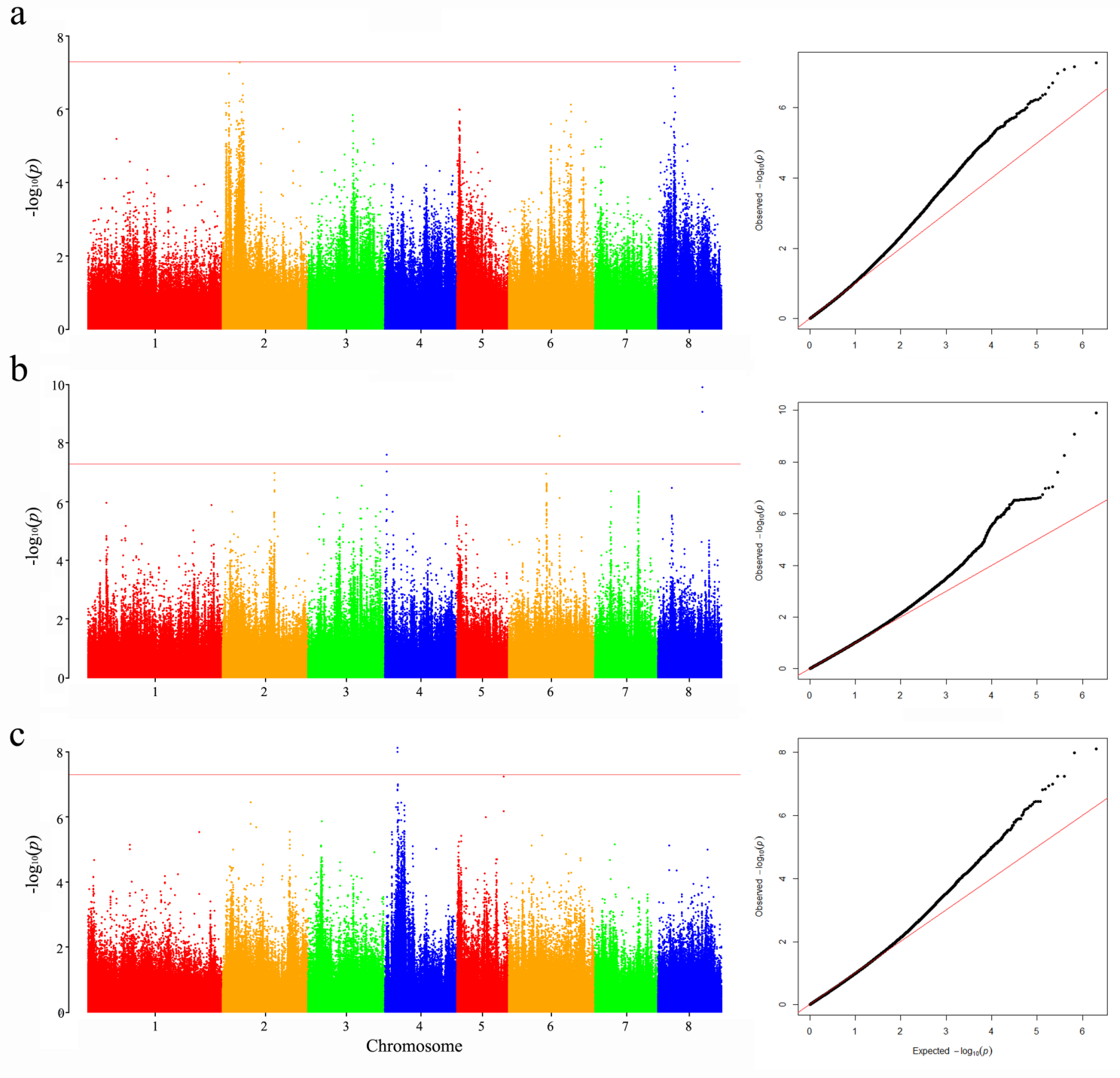


Figure S15. Genome-wide association study for citric acid content in fruit in (a) 2013, (b) 2014, and (c) 2015 for 313 landraces and improved varieties using FaSTLMM software.


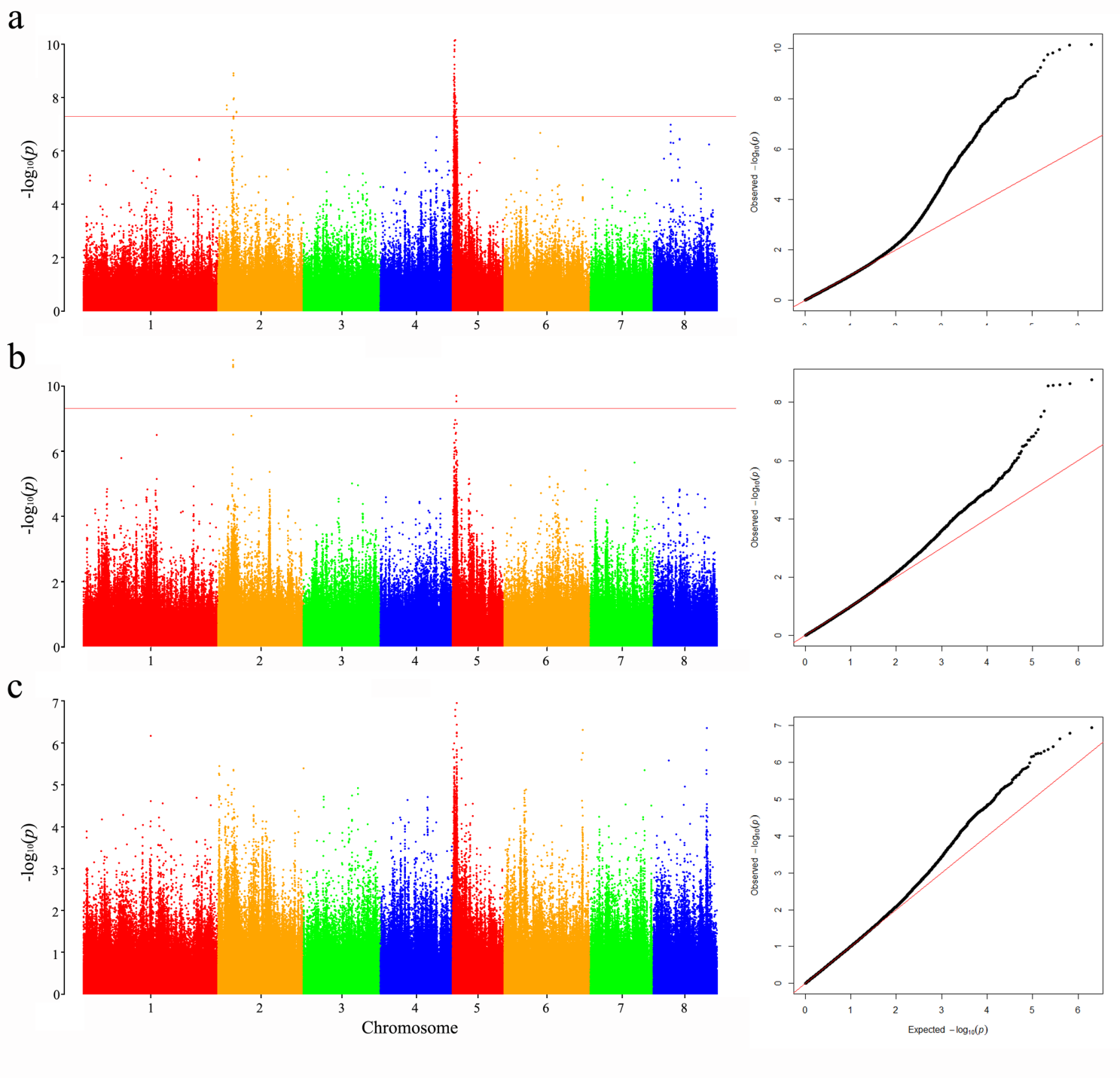


Figure S16. Genome-wide association study for malic acid content in fruit in (a) 2013, (b) 2014, and (c) 2015 for 313 landraces and improved varieties using FaSTLMM software.


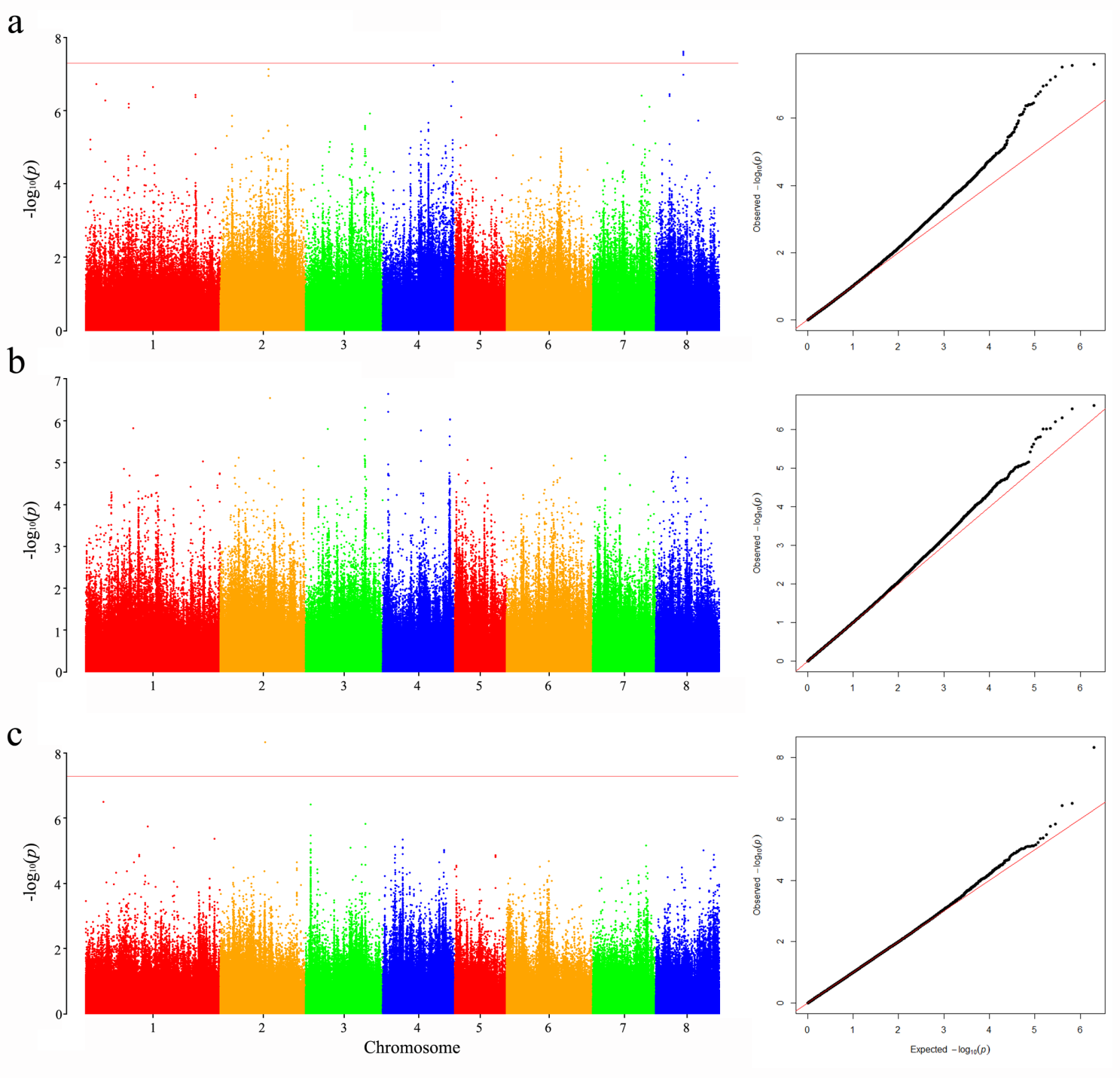


Figure S17. Genome-wide association study for quininic acid content in fruit in (a) 2013, (b) 2014, and (c) 2015 for 313 landraces and improved varieties using FaSTLMM software.


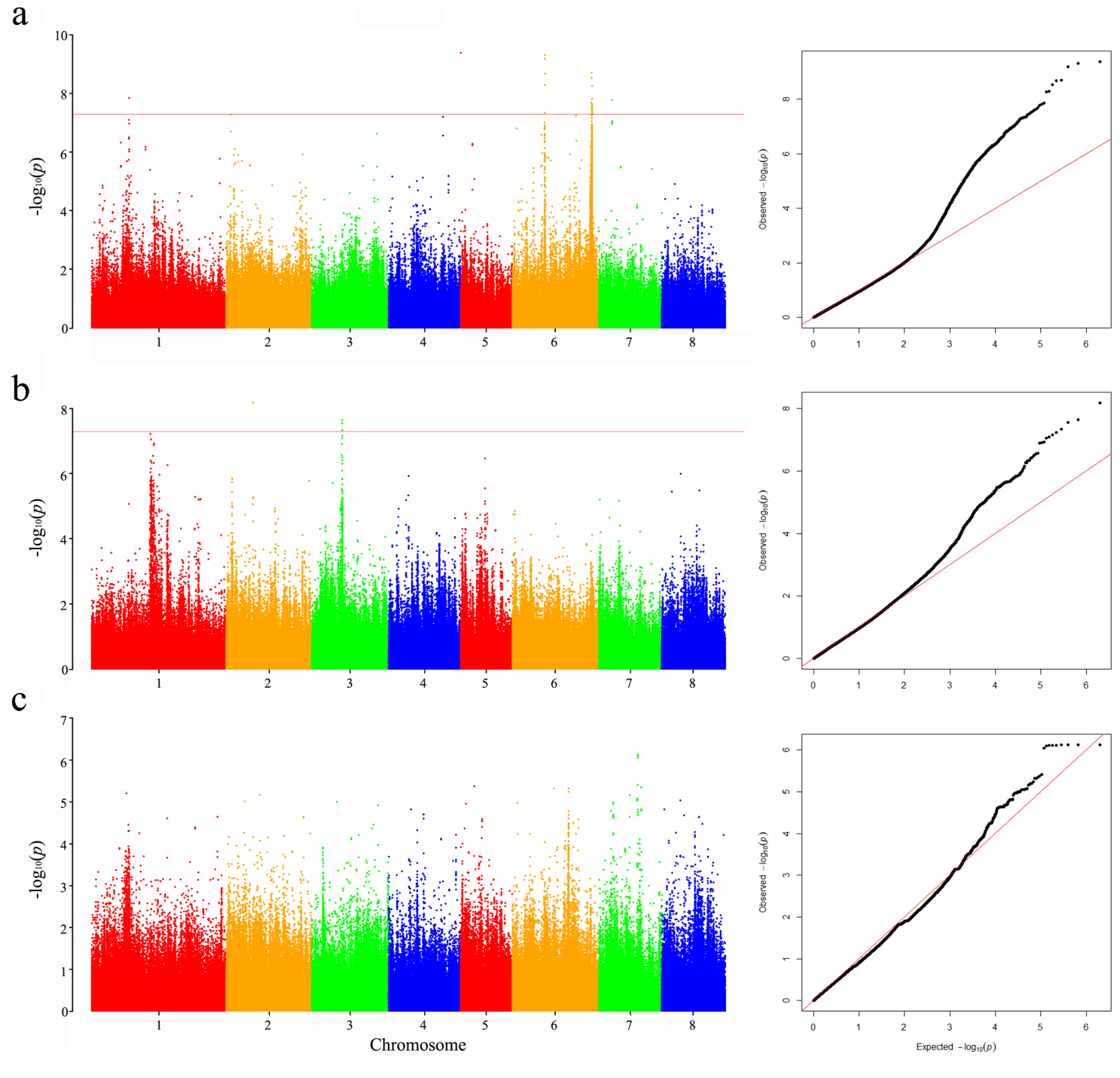


Figure S18. Genome-wide association study for succinic acid content in fruit in (a) 2013, (b) 2014, and (c) 2015 for 313 landraces and improved varieties using FaSTLMM software.


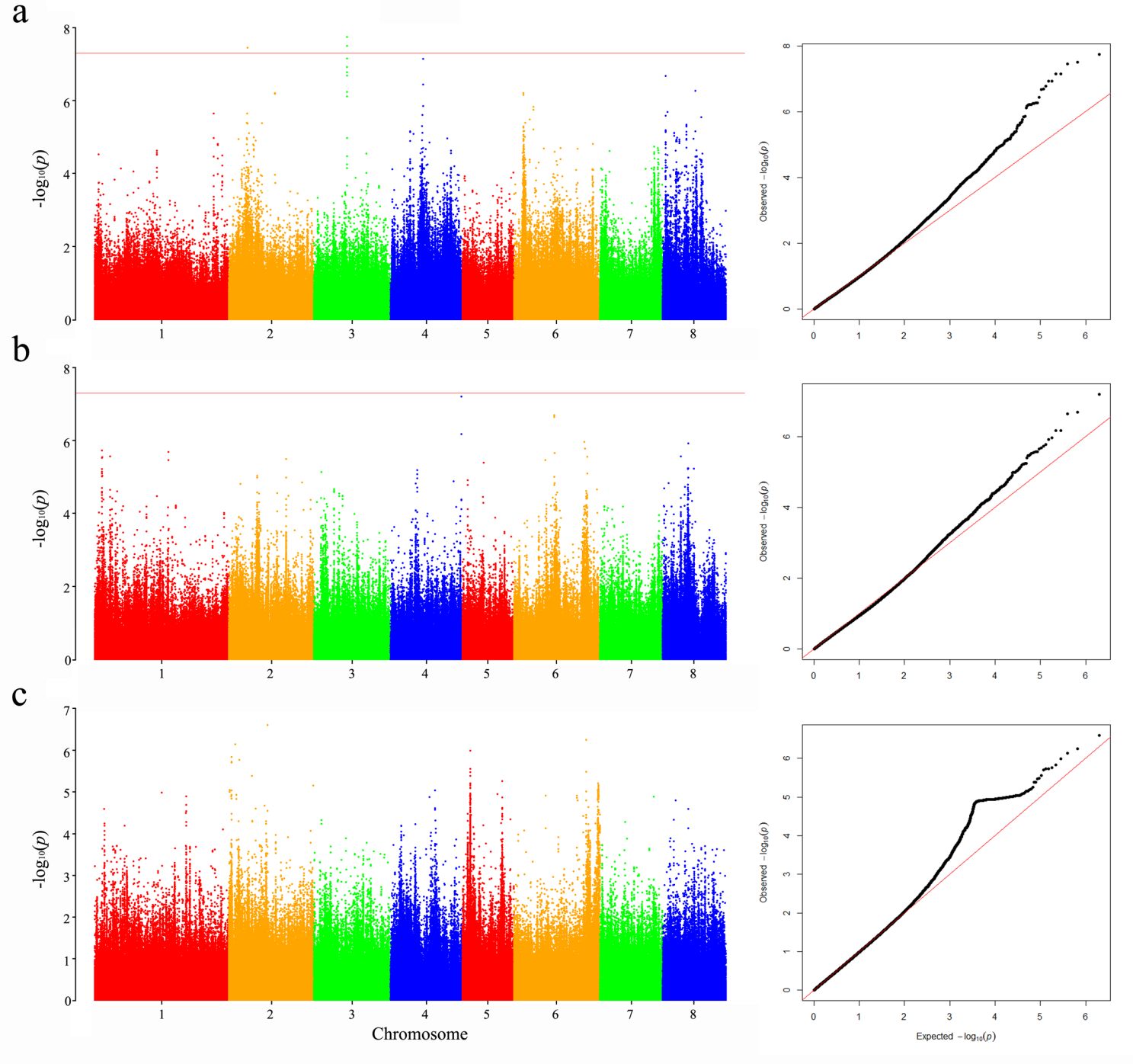


Figure S19. Genome-wide association study for fructose content in fruit in (a) 2013, (b) 2014, and (c) 2015 for 313 landraces and improved varieties using FaSTLMM software.


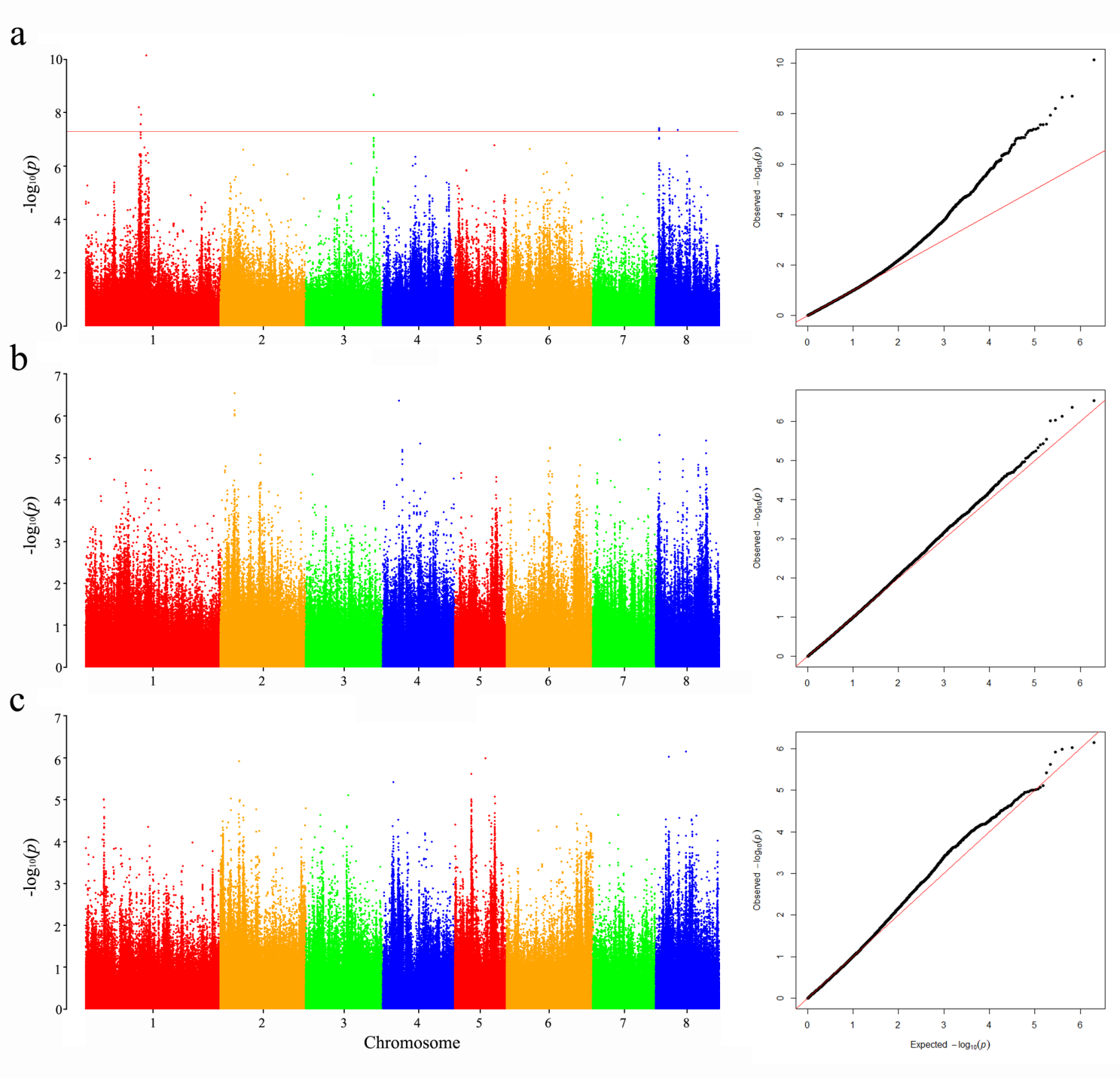


Figure S20. Genome-wide association study for glucose content in fruit in (a) 2013, (b) 2014, and (c) 2015 for 313 landraces and improved varieties using FaSTLMM software.


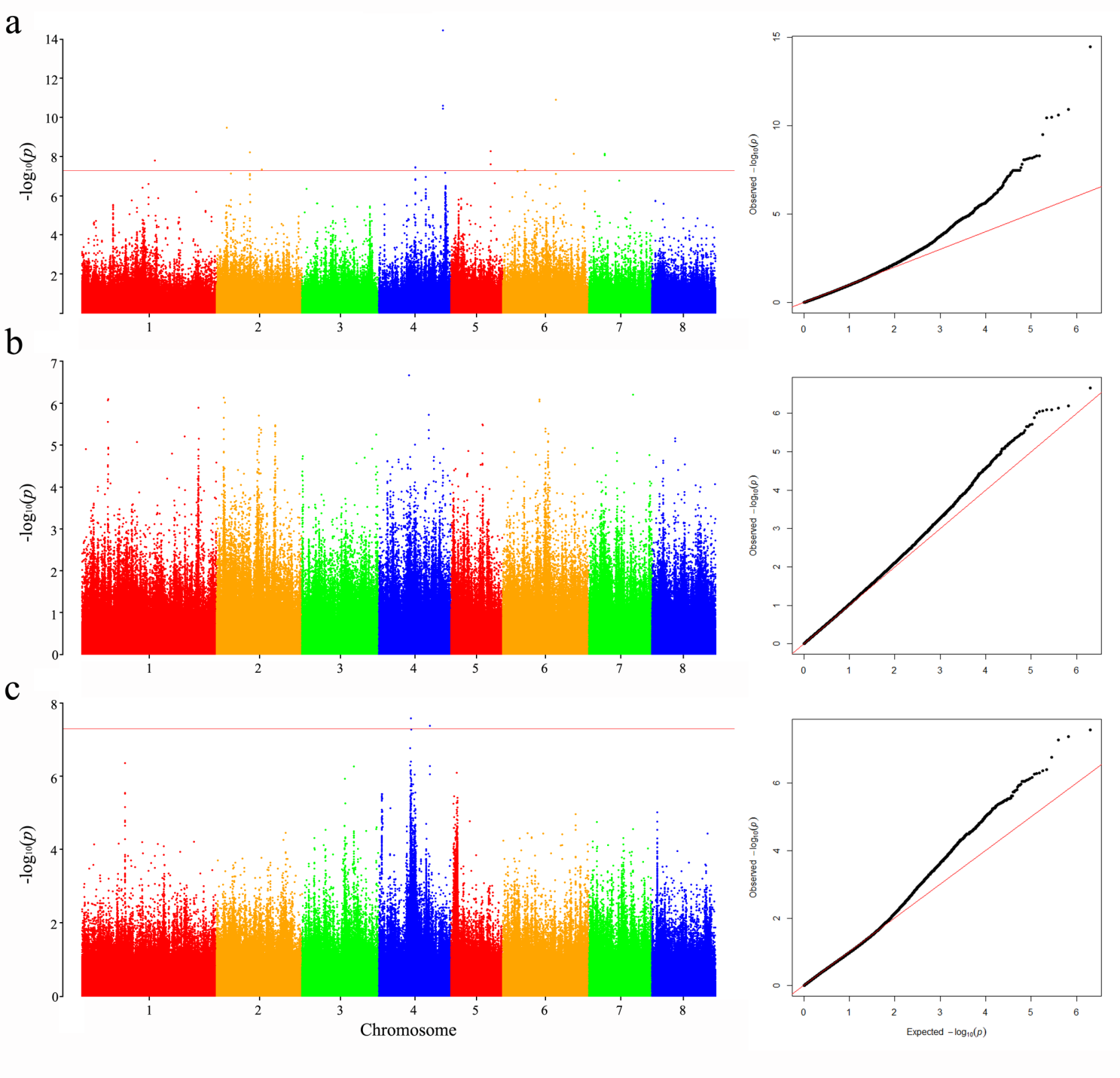


Figure S21. Genome-wide association study for sorbitol content in fruit in (a) 2013, (b) 2014, and (c) 2015 for 313 landraces and improved varieties using FaSTLMM software.


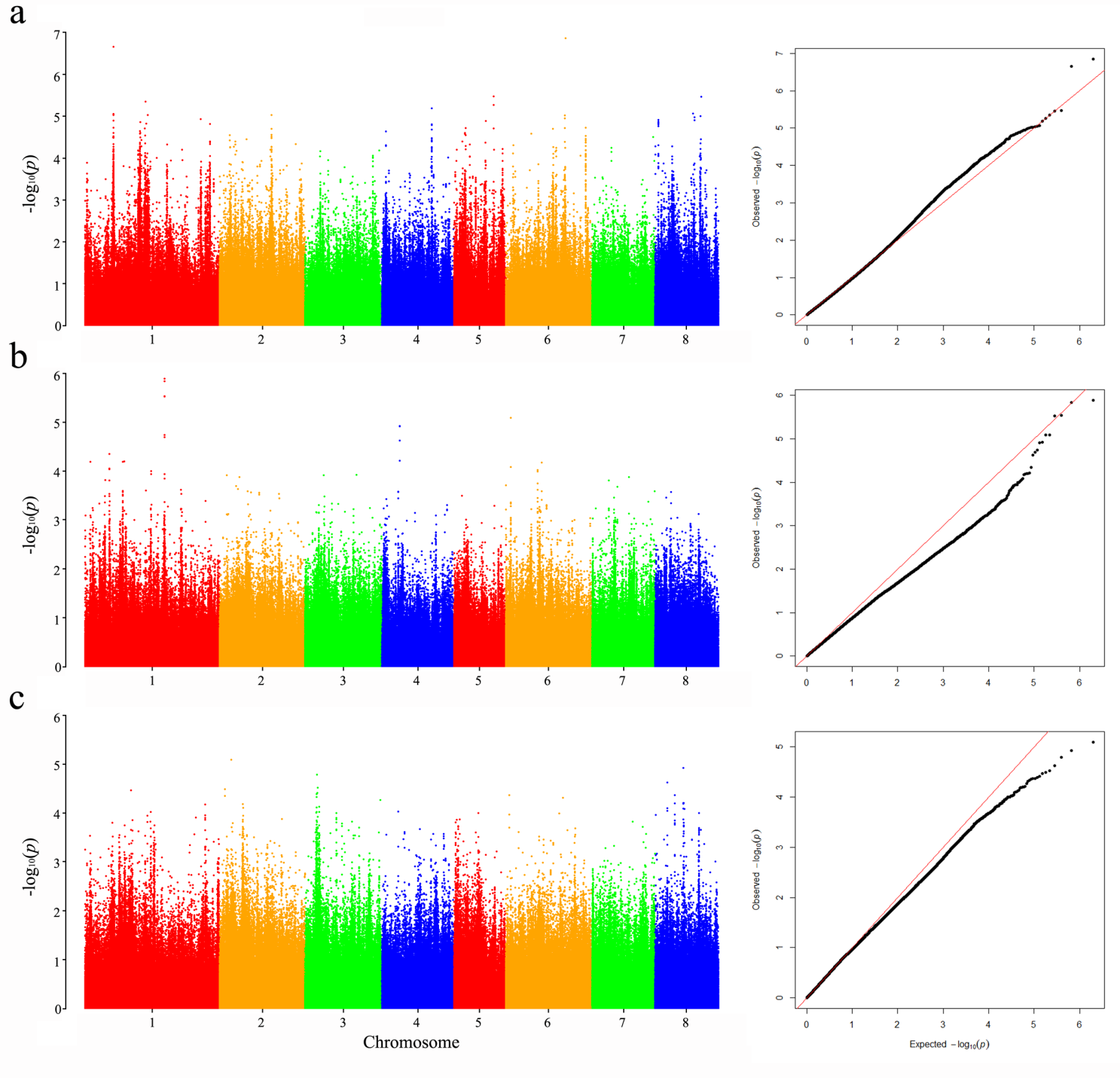


Figure S22. Genome-wide association study for sucrose content in fruit in (a) 2013, (b) 2014, and (c) 2015 for 313 landraces and improved varieties using FaSTLMM software.


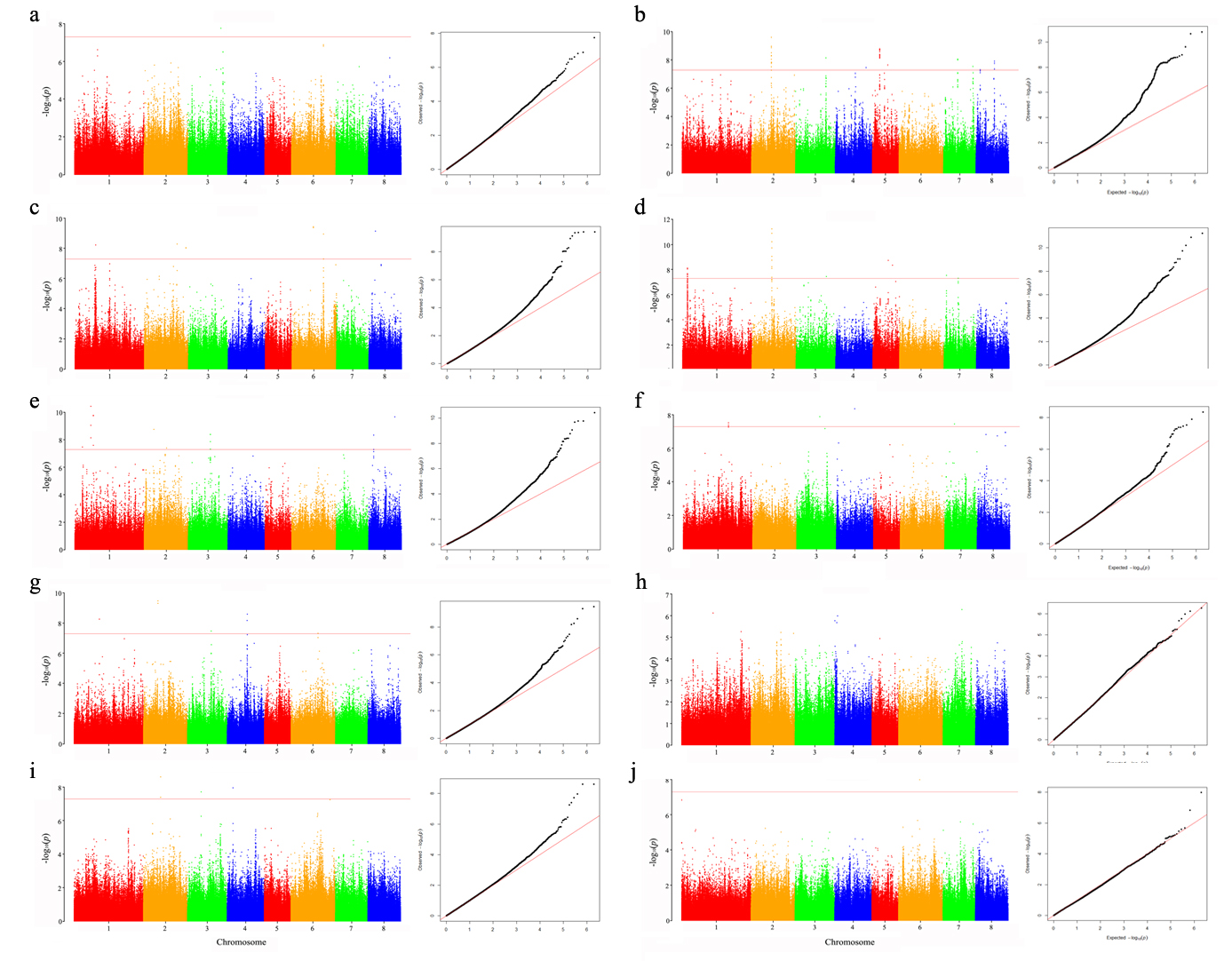


Figure S23. Genome-wide association study for catechin content in fruit in (a) 2014 and (b) 2015, epicatechin content in fruit in (c) 2014 and (d) 2015, chlorogenic content in fruit in (e) 2014 and (f) 2015, neochlorogenic content in fruit in (g) 2014 and (h) 2015, procyanidin B1 content in fruit in (i) 2014 and (j) 2015 for 313 landraces and improved varieties, which were performed using FaSTLMM software.


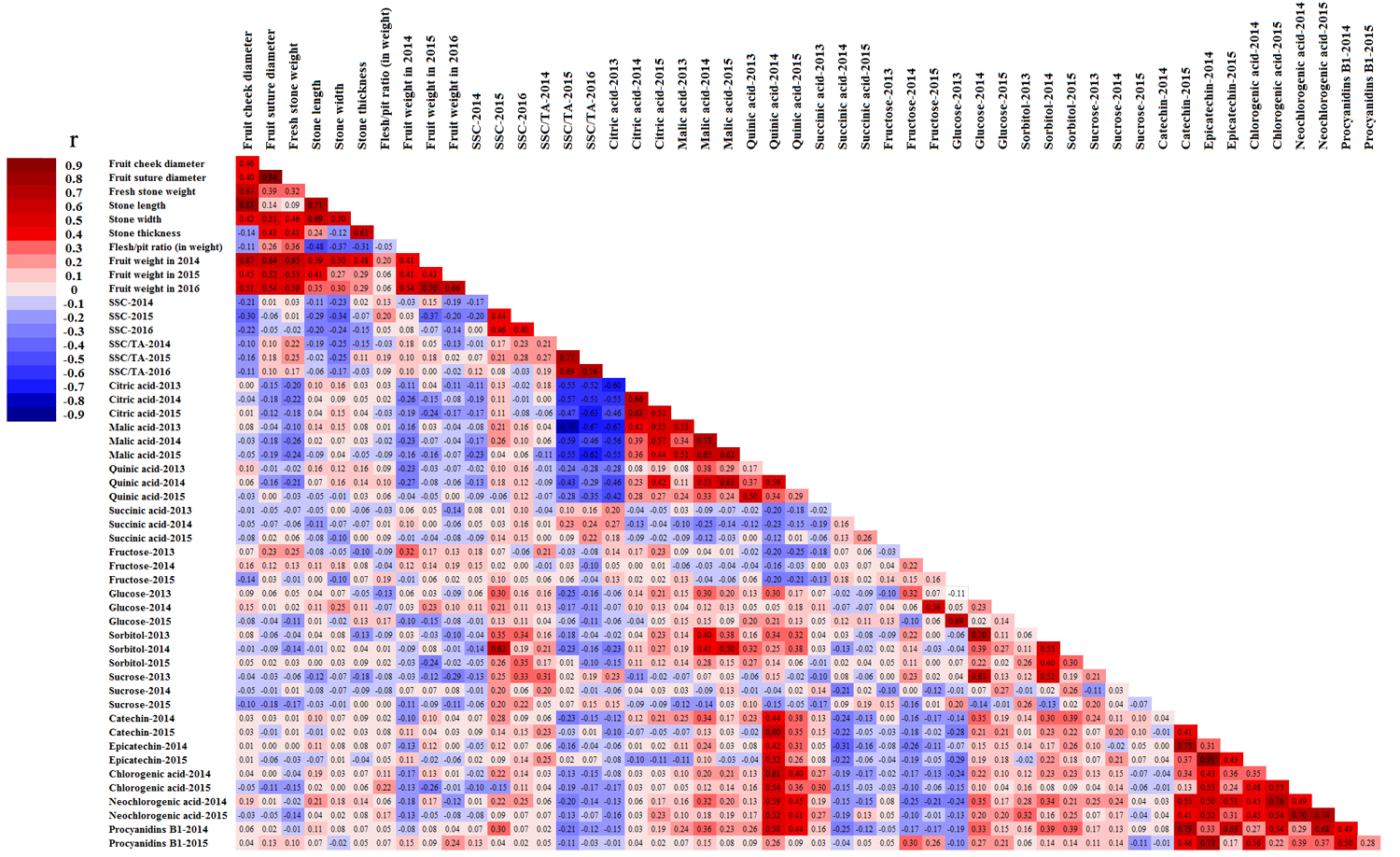


Figure S24. The pearman correlation coefficients calculated among the traits of peach evaluated in the study.


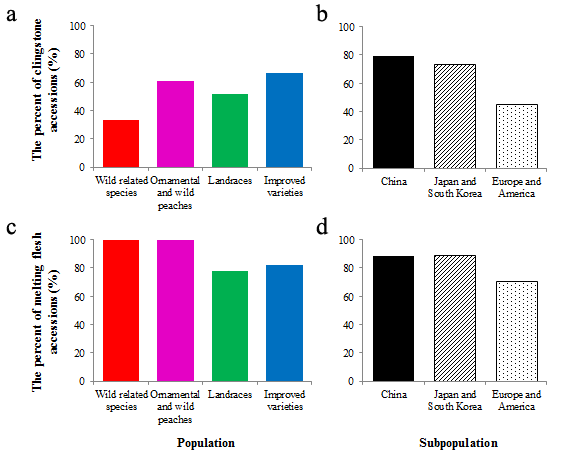


Figure S25. The phenotypic distribution of flesh adhesion (a) and texture (c) in different populations and subpopulations (b and d) of improved varieties.


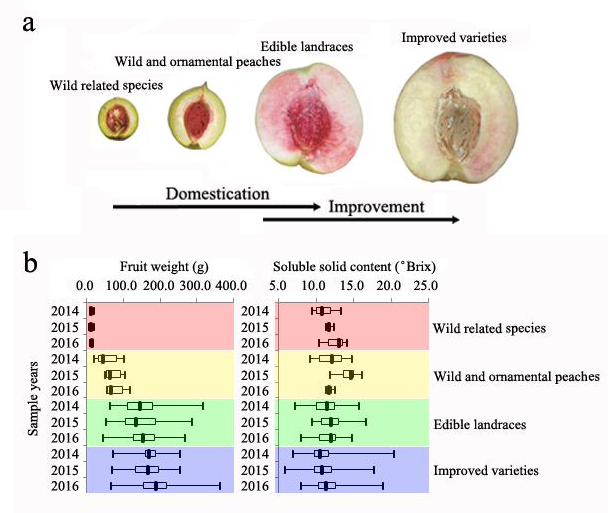


Figure S26. The evolution of peach with respect to fruit/stone size (a), fruit weight and soluble solids content (b). Data were derived from analysis of 323 accessions.


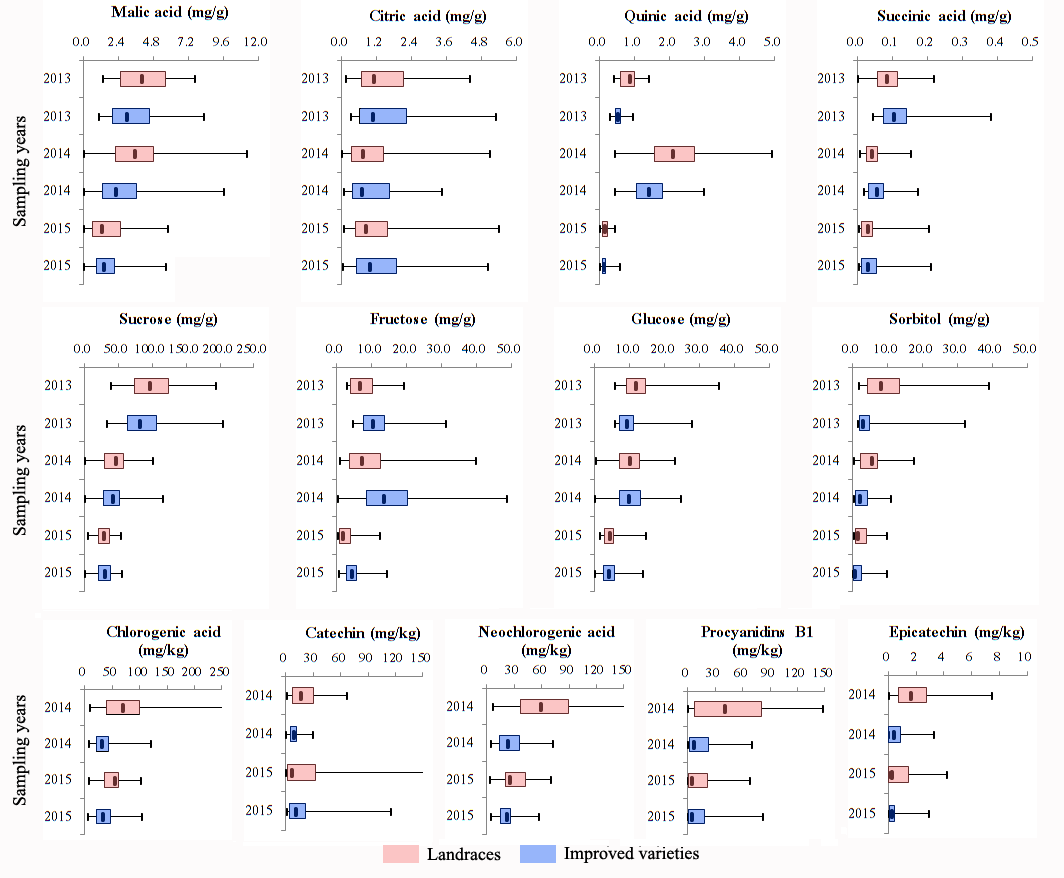


Figure S27. The content of 4 acid, 4 sugar, and 5 polyphenol related fruit compounds in landraces and improved varieties over different years of sampling.


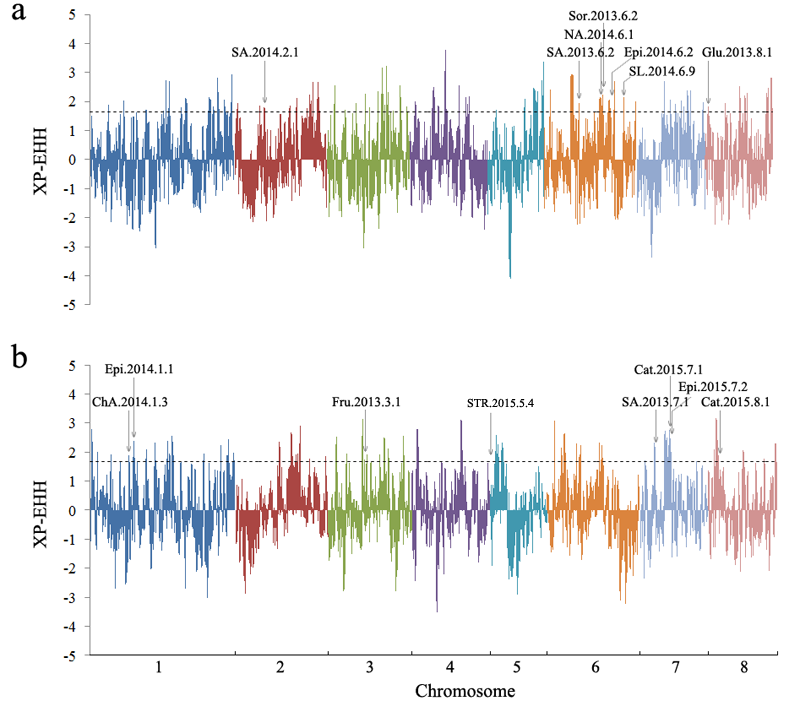


Figure S28. Results of the Cross Population Extended Haplotype Homozygosity (XP-EHH) analysis to detect selection sweeps under domestication (a) and improvement (b). In the panel, the dotted line represents the upper 5% quantile of XP-EHH values. The QTLs which overlapped with the selection sweeps are indicated with arrows.


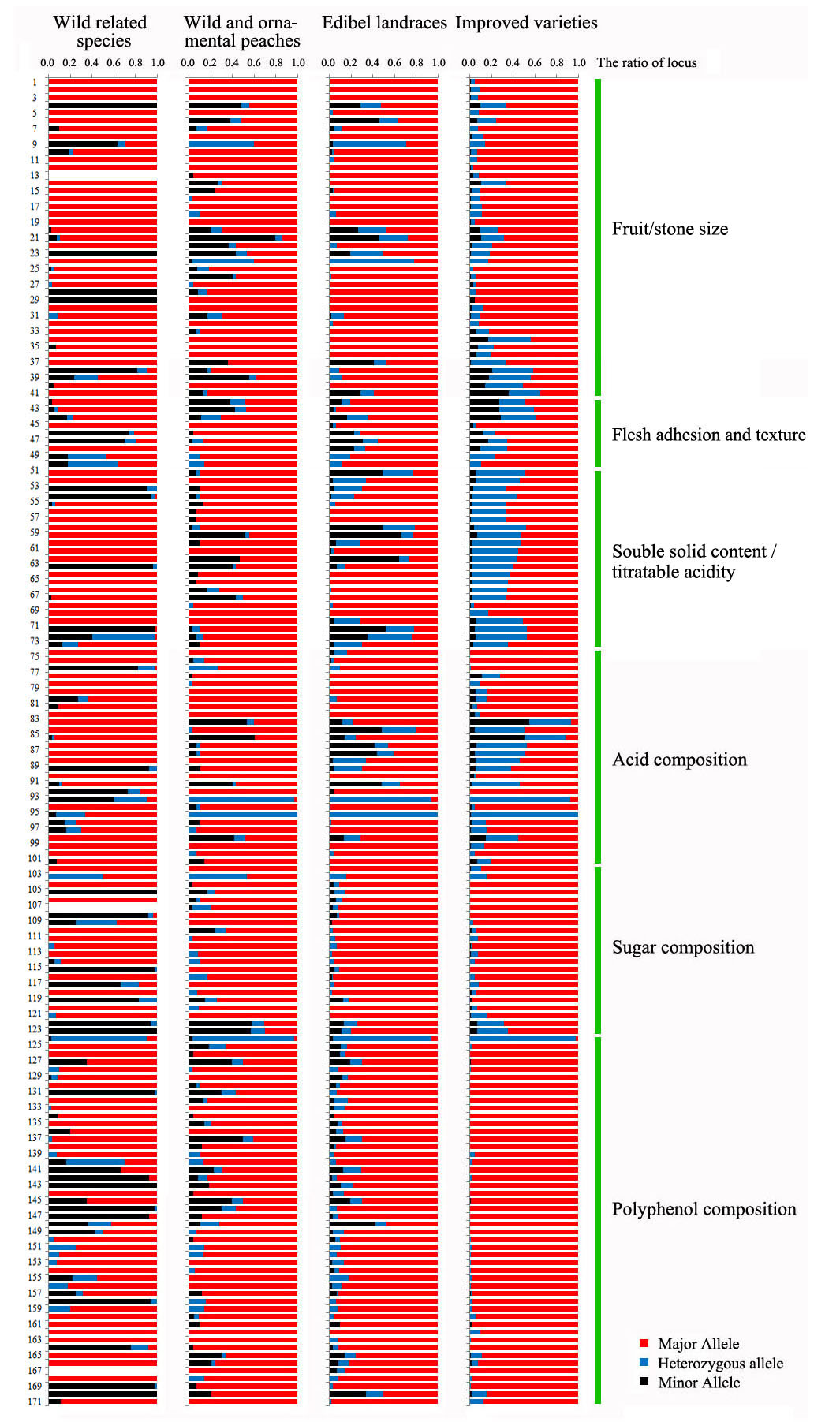


Figure S29. The change in allele frequency for the SNP most highly associated with a range of trait loci in different peach populations. The number in the left column indicates the locus accession in Table S11.
