# Supplementary material for: Comparative population genomics identified genomic regions and candidate genes associated with fruit domestication traits in peach
Source: Plant Biotechnol J. 2019 Apr 16;17(10):1954–70. doi: 10.1111/pbi.13112 (PMC6737019; doi:10.1111/pbi.13112)
Supplement: Supplementary file 3 — Appendix S1 Candidate genes identified in the sweep regions with the most obvious and large‐effect selection signals. [file PBI-17-1954-s004.docx]

**Candidate genes identified in the sweep regions with the most obvious selection signals.**

The region with the strongest putative selection for domestication (42.25-42.40 Mb of Chr. 1) (Figure 5c and Table S11) was identified by bothπ and XP-EHH values (Table S10) and comprises six resistance genes as well as the following genes involved indirectly in the resistance: *Prupe.1G513600* encoding ethylene-responsive transcription factor RAP2-4, *Prupe.1G513800* encoding probable trehalose-phosphate phosphatase, *Prupe.1G513900* encoding late embryogenesis abundant hydroxyproline-rich glycol protein, *Prupe.1G514500* encoding tetratricopeptide repeat containing thioredoxin, *Prupe.1G514600* encoding VQ motif-containing protein, and *Prupe.1G515200* encoding annexin D1. The relatively low diversity of resistance genes in the sweep is consistent with the lack of pest and disease resistance in *P. persica*, compared with its wild related species. The region is also close to a major Mendelian trait locus for resistance to green peach aphid, which was linked to a simple sequence repeat (SSR), UDP-022 (Chr1: 43,622,315 bp) (Lambert *et al.*, 2016). The strongest putative selection for improvement is located at the top of Chr. 5 between 1.50 to 1.65 Mbp (Figure 5e and Table S11) and was identified by overlapped π and XP-EHH values (Table S10). The region includes a gene that encodes a protein that regulates pollen tube growth and its position is close to the association signal for the non-acid fruit trait (0.5 Mb in Chr. 5, Cao *et al.*, 2014).

**The candidate genes in the large-effect sweep regions.**

We also identified several large-effect sweep regions with lengths equal to or longer than 200 kbp that may be the result of artificial selection during long-term evolution (Jia *et al.*, 2013). The block located at the bottom of Chr. 1 at 42.45 to 42.65 Mbp (Table S12), close to the regions with the strongest selection pressure for domestication (see above), may be related to missing resistance genes. Among the regions related to improvement (Table S12), we found more novel regions (seven blocks) identified by both π and XP-EHH values distributed among Chr. 2 (14.3-14.5 Mbp), 5 (1.75-2.00 Mbp; 2.30-2.55 Mbp; 3.85-4.05 Mbp), 6 (5.60-5.80 Mbp; 6.00-6.20 Mbp), and 8 (2.60-2.85 Mbp). Of particular interest was a large-effect sweep region with low diversity on Chr. 5 from 1.75 to 4.05 Mbp, which was just downstream of the locus controlling non-acid fruit (541,075 bp in Cao et al., 2016 annotated in peach genome V1.0, corresponding to 541,076 bp in peach genome V2.0) and to QTLs controlling soluble solids content (SSC), fructose, glucose, sorbitol, sucrose, and total sugar (Cirilli *et al.*, 2016; Mora *et al.*, 2017).

**Supplementary References**

Cao, K., Zheng, Z.J., Wang, L.R., Liu, X., Zhu, G.R., Fang, W.C., Chen, S.F., et al. (2014). Comparative population genomics reveals the domestication history of the peach, *Prunus persica*, and human influences on perennial fruit crops. *Genome Biol.* **15**:415.

Cao, K., Zhou, Z.K., Wang, Q., Guo, J., Zhao, P., Zhu, G.R., Fang, W.C., et al. (2016). Genome-wide association study of 12 agronomic traits in peach. *Nat. Commun.* **7**:13246.

Cirilli, M., Bassi, D., and Ciacciulli, A. (2016). Sugars in peach fruit: a breeding perspective. *Hortic. Res.* **3**:15067.

Jia, G.Q., Huang, X.H., Zhi, H., Zhao, Y., Zhao, Q., Li, W.J., Chai, Y., et al. (2013). A haplotype map of genomic variations and genome-wide association studies of agronomic traits in foxtail millet (*Setaria italica*). *Nat. Genet.* **45**:957-961.

Lambert, P., Campoy, J.A., Pacheco, I., Mauroux, J., Da Silva Linge, C., Micheletti, D., Bassi, D., et al. (2016). Identifying SNP markers tightly associated with six major genes in peach [*Prunus persica* (L.) Batsch] using a high-density SNP array with an objective of marker-assisted selection (MAS). *Tree Genet. Genomes* **12**:121.

Mora, J.R.H., Micheletti, D., Bink, M., de Weg, E.V., Cantín, C., Nazzicari, N., Caprera, A., et al. (2017). Integrated QTL detection for key breeding traits in multiple peach progenies, *BMC Genomics* **18**:404.
